# Activation of the Type III Secretion System of Enteropathogenic Escherichia coli Leads to Remodeling of Its Membrane Composition and Function

**DOI:** 10.1128/msystems.00202-22

**Published:** 2022-04-28

**Authors:** Anish Zacharia, Ritesh Ranjan Pal, Naama Katsowich, Chanchal Thomas Mannully, Aida ibrahim, Sivan Alfandary, Raphael Serruya, Amit K. Baidya, Sigal Ben-Yehuda, Ilan Rosenshine, Arieh Moussaieff

**Affiliations:** a The Institute for Drug Research, The Hebrew University of Jerusalem, Jerusalem, Israel; b Department of Microbiology and Molecular Genetics, Institute of Medical Research Israel-Canada, Faculty of Medicine, The Hebrew University of Jerusalem, Jerusalem, Israel; c School of Biological Sciences, Indian Association for the Cultivation of Science, Kolkata, India; d Molecular Biophysics Unit, Indian Institute of Science, Bangalore, India; Vanderbilt University Medical Center

**Keywords:** CesT, CsrA, lipid metabolism, lipidomics, terpenoids, type III secretion system, cell envelope, enteropathogenic *E. coli*, isoprenoids, phospholipids

## Abstract

The cell envelope of Gram-negative bacteria is a complex structure, essential for bacterial survival and for resistance to many antibiotics. Channels that cross the bacterial envelope and the host cell membrane form secretion systems that are activated upon attachment to host, enabling bacteria to inject effector molecules into the host cell, required for bacterium-host interaction. The type III secretion system (T3SS) is critical for the virulence of several pathogenic bacteria, including enteropathogenic Escherichia coli (EPEC). EPEC T3SS activation is associated with repression of carbon storage regulator (CsrA), resulting in gene expression remodeling, which is known to affect EPEC central carbon metabolism and contributes to the adaptation to a cell-adherent lifestyle in a poorly understood manner. We reasoned that the changes in the bacterial envelope upon attachment to the host and the activation of a secretion system may involve a modification of the lipid composition of bacterial envelope. Accordingly, we performed a lipidomics analysis on mutant strains that simulate T3SS activation. We saw a shift in glycerophospholipid metabolism toward the formation of lysophospholipids, attributed to corresponding upregulation of the phospholipase gene *pldA* and the acyltransferase gene *ygiH* upon T3SS activation in EPEC. We also detected a shift from menaquinones and ubiquinones to undecaprenyl lipids, concomitant with abnormal synthesis of O antigen. The remodeling of lipid metabolism is mediated by CsrA and associated with increased bacterial cell size and zeta potential and a corresponding alteration in EPEC permeability to vancomycin, increasing the sensitivity of T3SS-activated strains and of adherent wild-type EPEC to the antibiotic.

**IMPORTANCE** The characterization of EPEC membrane lipid metabolism upon attachment to the host is an important step toward a better understanding the shift of EPEC, a notable human pathogen, from a planktonic to adherent lifestyle. It may also apply to other pathogenic bacteria that use this secretion system. We predict that upon attachment to host cells, the lipid remodeling upon T3SS activation contributes to bacterial fitness and promotes host colonization, and we show that it is associated with increased cell permeability and higher sensitivity to vancomycin. To the best of our knowledge, this is the first demonstration of a bacterial lipid remodeling due to activation of a secretion system.

## INTRODUCTION

The cell envelope of Gram-negative bacteria is a complex structure, consisting of a bilayered plasma membrane, a periplasm, and an outer membrane (1). This complex is essential for bacterial survival in harsh environments and for resistance to many antibiotics (2). Remarkably, channels that cross the bacterial envelope and the host cell membrane are regulated upon attachment to host, enabling bacteria to inject effector proteins into the host cell, critical for the bacterium-host interaction (3).

Enteropathogenic Escherichia coli (EPEC) is a common cause of pediatric diarrhea (4). Upon attachment to the host intestinal epithelium, this pathogen employs a type III secretion system (T3SS) to inject effector proteins into the host cells. The T3SS is encoded within a pathogenicity island termed the locus of enterocyte effacement (LEE) (5), composed of a cluster of transcriptional units containing 41 genes encoding T3SS structural components, six translocated effectors, and related proteins (6, 7). The LEE5 operon contains three genes: *tir*, *cesT*, and *eae*, encoding Tir (translocated intimin receptor), CesT, and intimin, respectively (8, 9). Tir is the most abundant effector and the first to be translocated into the host cell (10, 11). CesT is a homodimer chaperone associated with many effectors, of which Tir is the major one (12, 13). CesT binds to two regions in Tir at the N and C termini through a specific recognition motif, promoting Tir stability and its translocation to the host (12). Intimin, the third product of the LEE5 operon, is an outer membrane protein that promotes adherence of EPEC to the host via interaction with the surface-exposed loop of translocated Tir (14, 15). This attachment type was termed intimate attachment (14).

CesT-Tir interaction is essential for Tir translocation into the host and the subsequent intimate attachment action. The second function of CesT-Tir interaction is related to the rearrangement of gene expression upon EPEC-host contact and the consequent Tir translocation (16). In planktonic EPEC, CesT remains bound to Tir and other effectors and the T3SS is not active but fully assembled, ready to immediately translocate the effectors into the host (17).

**FIG 1:**
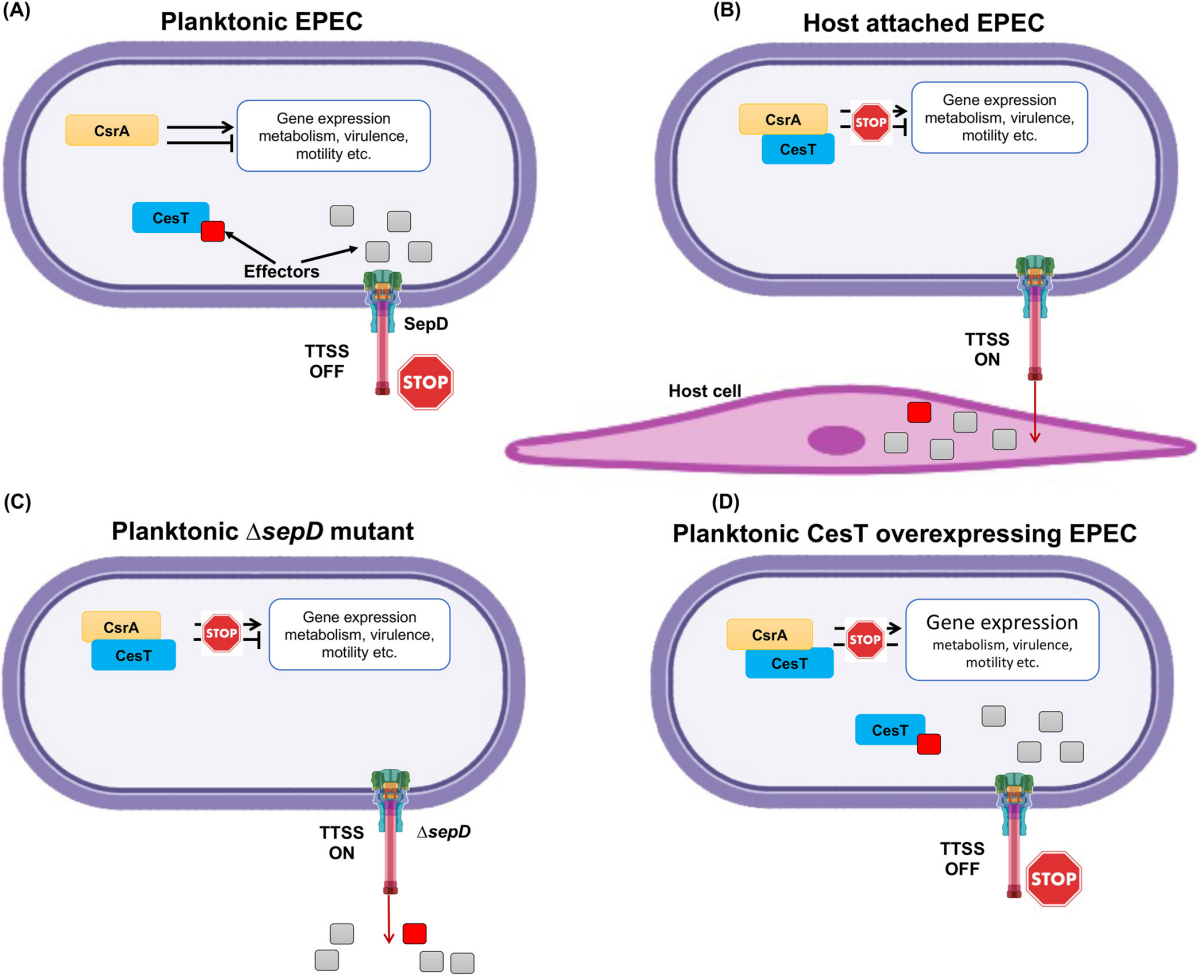
A scheme of the T3SS regulation of EPEC attachment to the host cell. To mimic the attached state of EPEC and study concomitant metabolic shifts, we used T3SS-regulated strains as follows. (A) Planktonic wild-type EPEC: the T3SS is not active. The effectors accumulate in the cytoplasm, and some of them (indicated as a red square) bind to CesT and sequester it. In these bacteria, CsrA is free to bind mRNA and regulate gene expression. (B) Host-attached wild-type EPEC: the T3SS is activated upon contact with the host, eliminating the effectors from the EPEC cytoplasm by injecting them into the host cell. The liberated CesT then binds to CsrA to inhibit CsrA-mRNA interaction. (C) Planktonic Δ*sepD* mutant: the T3SS is constitutively active, and effectors are secreted. The liberated CesT binds to CsrA and inhibits CsrA-mRNA interaction. (D) Planktonic CesT-overexpressing EPEC: CesT is expressed at levels that allow binding to all the CesT-binding effectors and CsrA, resulting in inhibition of CsrA-mRNA interactions regardless of T3SS activity.

The secretory activity of T3SS is activated only upon attachment to the host cell and insertion of the translocon (i.e., the EspBD channel) to the host cell membrane. Three proteins, termed “switch proteins,” form a complex that is responsible to prevent effector secretion in nonattached EPEC and promote secretion upon translocon insertion. How this switch complex works is not fully understood. SepD is one of the switch proteins, and in its absence, the switch complex can no longer prevent secretion via the T3SS into the medium regardless of the host cell. Thus, the SepD mutant but not the wild type (WT) mimics the T3SS activation and consequent effector secretion, leading to liberation of CesT and thus a higher level of free CesT (7, 11, 12, 17–20). The delivery of these effectors into the host liberates CesT, resulting in increased levels of free CesT in the EPEC cytoplasm. The liberated CesT then interacts with an alternative binding partner, the carbon storage regulator A (CsrA) (12, 16, 21). CsrA is an RNA-binding protein and posttranscriptional regulator which coordinates numerous bacterial functions, including motility, metabolism, and virulence (22, 23). Notably, the elevated levels of free CesT, upon effector injection, competitively inhibit CsrA-mRNA interaction (12, 16, 21). Since CsrA binds to the mRNA of numerous genes and regulates the stability and/or translation of these mRNAs, CesT-CsrA interaction results in remodeling of gene expression (Fig. 1).

Importantly, while the wild type and *sepD* mutant have similar total CesT levels, the levels of free CesT vary. Consequently, the levels of CsrA inhibition by CesT also vary between the two strains (16, 24). As to the CesT-overexpressing strain, this strain has higher levels of total CesT and thus higher levels of free CesT regardless of host cell or secretion of effectors.

The significance of the CesT-CsrA switch to the bacterial physiology can be partially extrapolated from studies comparing wild-type E. coli to *csrA* mutants (22, 25–28). However, *csrA* mutants are not an ideal model to mimic host interacting EPEC since the CesT-CsrA interaction induces only partial and temporal CsrA inhibition. Previous reports point to alternative approaches that mimic the physiological state of host-attached EPEC. The first approach employs the EPEC *sepD* mutant (Δ*sepD*), which expresses the T3SS, which constitutively secretes Tir regardless of host attachment (16, 29). Thus, the *sepD* mutant contains higher levels of liberated CesT, which is free to interact with CsrA. Another approach uses wild-type EPEC containing a plasmid that expresses CesT under the control of an isopropyl-d-thiogalactopyranoside (IPTG)-regulated promoter (WT/pCesT). Upon IPTG treatment it overexpresses CesT, which readily interacts with CsrA (Fig. 1).

In this study, we combined lipidomics and genetic methodologies to characterize the shift of EPEC metabolism under conditions that mimic infection and the associated CsrA inhibition. We sought to unveil possible shifts in membrane lipid metabolism that take place upon secretion system activation, by an unbiased lipidomics analysis. However, obtaining biological material from injecting EPEC in quantities that allow lipidomics analysis is challenging, as only a small subpopulation is engaged in injection, while in the rest of the population that is not in direct contact with the host, the T3SS remains inactive (11). To overcome this hurdle and obtain uniform populations, we compared wild-type EPEC that does not secrete Tir in a planktonic state (thus, Tir sequesters CesT) to an isogenic EPEC Δ*sepD* mutant that constitutively secretes Tir and, consequently, CesT is liberated, free to interact with CsrA.

Our lipidomics analysis pointed to a major shift from planktonic to adherent bacteria lipidome. We expect this lipid shift to be a general trait for secretion systems, in particular in E. coli spp. experiencing CsrA inhibition. We assume that this lipid modulation is involved in the adaptation of EPEC to the cell-adherent lifestyle.

10.1128/msystems.00202-22.1 FIG S1 Schematic representation of the lipid classes modified by T3SS activation in EPEC. (A) Glycerophospholipid structure representing glycerol unit attached to the two fatty acyl chains (R1 and R2) or single acyl chain (LysoPL). A phosphate head group determines the lipid class and is represented by X. (X-i) Phosphatidylethanolamine (PE); (X-ii) phosphatidylglycerol (PG); (X-iii) phosphatidyl serine. (B) Cardiolipin (CL) is a phospholipid with two phosphatidic acids linked to the two carbons of glycerol unit; hence, four acyl chains (R1 plus R2 and R3 plus R4) are attached to the respective glycerol units. (C) Monomeric isoprene unit with five carbons. These isoprenes form different number of repeats (represented as n) and may bind to diphosphate (Y-i), ubiquinone (Y-ii), and menaquinone(Y-iii). Download FIG S1, TIF file, 2.0 MB. Copyright © 2022 Zacharia et al. 2022 Zacharia et al. https://creativecommons.org/licenses/by/4.0/ This content is distributed under the terms of the Creative Commons Attribution 4.0 International license.

## RESULTS

We used EPEC strains that mimic the host-attached status to study the changes in their lipid metabolism. We compared wild-type EPEC that does not secrete Tir in a planktonic state (thus, Tir sequesters CesT) to an isogenic EPEC Δ*sepD* mutant that constitutively secretes Tir and, consequently, CesT is liberated, free to interact with CsrA. To verify the CsrA-mediated regulation of metabolism in these strains, we used EPEC with a deleted *csrA* gene (Δ*csrA*). The latter was also used as a reference to available data of the metabolic profile of the EPEC and E. coli K-12 Δ*csrA* mutant (27, 30, 31). We grew the bacteria under conditions optimal for the expression of the T3SS genes (i.e., growth in 500 mL of Dulbecco’s modified Eagle medium [DMEM] at 37°C without shaking to an optical density at 600 nm [OD_600_] of 0.6). Under these experimental conditions, Δ*csrA* strains showed an attenuated growth rate (Fig. S2), in line with previous literature (23, 27, 31). The Δ*sepD* Δ*cesT* double mutant showed a mild attenuation in growth rate, whereas the Δ*sepD* strain and EPEC supplemented with CesT-expressing plasmid (WT/pCesT) showed growth rates similar to that of the wild type.

10.1128/msystems.00202-22.2 FIG S2 Growth curve of EPEC wild-type, Δ*sepD*, Δ*csrA*, Δ*sepD* Δ*cesT*, and WT/pCesT strains. EPEC strains were grown overnight in LB broth and then cultured in high-glucose DMEM (lacking glutamine and pyruvate) at 37°C under static conditions, until reaching an optical density at 600 nm (OD_600_) of 0.6, at which point bacterial growth was determined. Download FIG S2, TIF file, 0.1 MB. Copyright © 2022 Zacharia et al. 2022 Zacharia et al. https://creativecommons.org/licenses/by/4.0/ This content is distributed under the terms of the Creative Commons Attribution 4.0 International license.

### CesT-CsrA interaction confers alterations in phospholipid (PL) and terpenoid-quinone pathways.

To study potential changes in EPEC lipid composition following T3SS activation, we performed a lipidomics analysis. We focused on the Δ*sepD* strain and used the WT/pCesT strain, overexpressing CesT, to confirm that the changes seen in the Δ*sepD* strain are CesT dependent. A Δ*sepD* Δ*cesT* double mutant, assumed to suppress the Δ*sepD* mutation, was also used for further confirmation.

**FIG 2:**
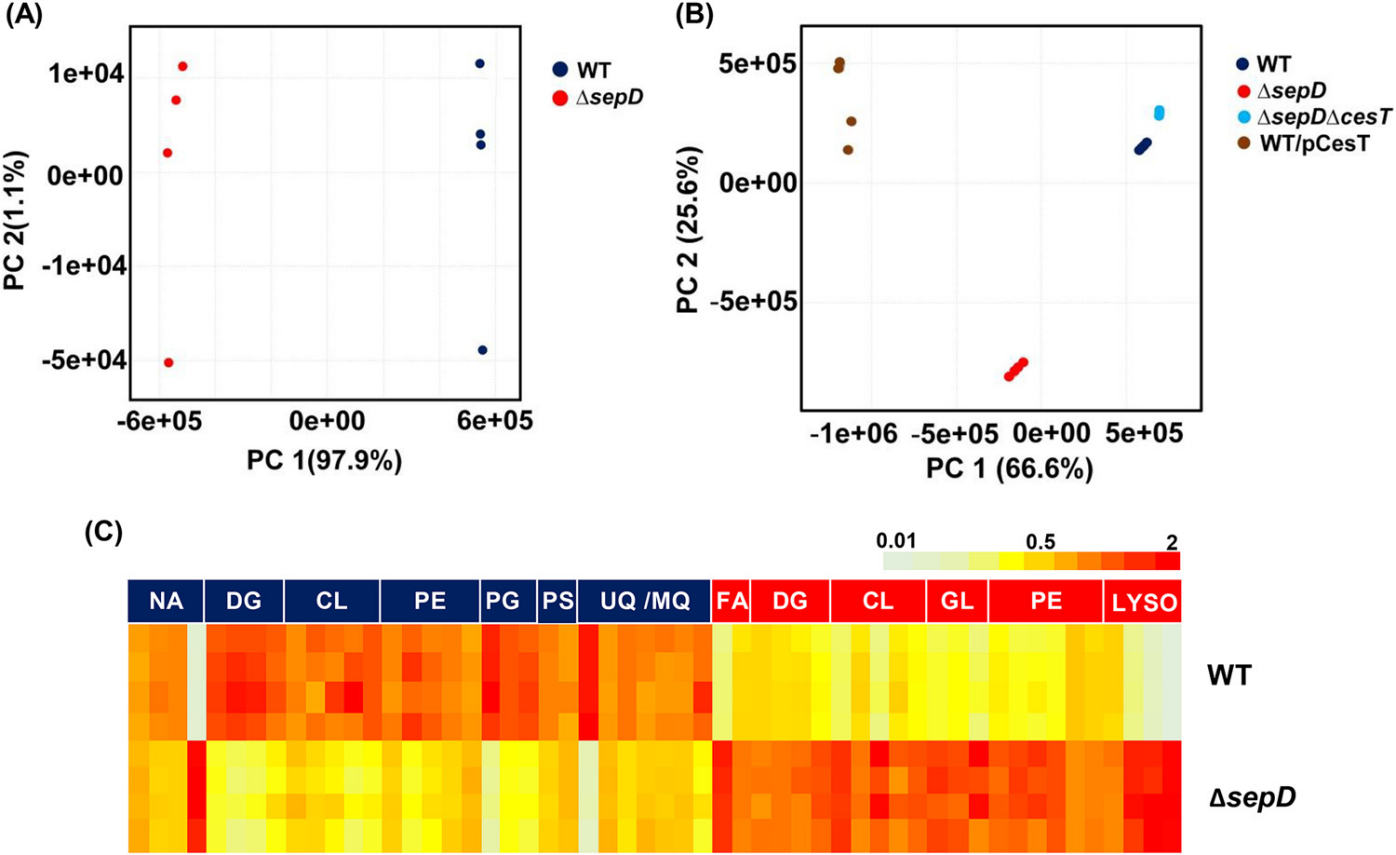
Levels of free CesT correlate with a profound effect on EPEC lipid metabolism. (A) Principal-component analysis (PCA) of lipids in wild-type (WT) versus ΔsepD EPEC (*n* = 4). (B) PCA of the lipidome of WT, Δ*sepD*, Δ*sepD* Δ*cesT*, and WT/pCesT strains. (C) A heat map of the differential lipids (variable importance in projection [VIP] >1) in *sepD* mutants, divided into lipid subclasses. Lipid subclasses with upregulated accumulation in mutants are in red, and lipid subclasses with downregulated accumulation in mutants are in blue. The color in each column indicates the fold change from mean abundance of the particular feature across all samples. Lyso, lysophospholipids; GL, glycolipids; CL, cardiolipins; DG, diacylglycerols; FA, fatty acids; Q/MK, ubiquinone/menaquinone; PE, phosphatidylethanolamines; PG, phosphatidylglycerols; PS, phosphatidylserines; NA, not assigned.

15,827 metabolic features were detected. After the exclusion of possible artifact features (see Materials and Methods), downstream lipidomics analysis was carried out using 3,277 metabolic features. We first analyzed the lipid composition of the wild type versus the *sepD* mutant. Principal-component analysis (PCA) demonstrated a striking separation between the lipidome of the Δ*sepD* mutant and that of wild-type EPEC, with PC1 accounting for 97.9% of the variance (Fig. 2A), suggesting that the increased levels of free CesT in the Δ*sepD* mutant induced a considerable shift in lipid metabolism. To test this hypothesis, we compared the lipidome of wild-type EPEC and the *sepD* mutant to that of the EPEC Δ*sepD* Δ*cesT* double mutant. The PCA of the lipidome of Δ*sepD* Δ*cesT* EPEC showed no shift in the lipid composition compared to the wild-type strain (Fig. 2B), indicating that CesT is required for inducing the shift in the lipid composition. To confirm this premise, we tested wild-type EPEC overexpressing CesT (WT/pCesT). In this case, the overexpression of CesT was sufficient for inducing a shift in lipid composition. This shift was more pronounced than that in the Δ*sepD* mutant (Fig. 2B, principal component 1 of the PCA), likely due to higher levels of free CesT in the overexpressing bacteria than in the Δ*sepD* mutant. Taken together, our data suggest that an increase in the levels of CesT is necessary and sufficient to induce a considerable shift in the lipid composition of EPEC.

A variable importance in projection (VIP) analysis of Δ*sepD* versus wild-type EPEC lipidome revealed 54 differential (VIP > 1) mass features. PLs and isoprenoids of the quinone terpenoid subclass were predominantly discriminant features in this analysis (Fig. 2C). Phosphatidylethanolamine (PE) and phosphatidylglycerol (PG) are the major lipid components of bacterial membranes. The inner membrane of Escherichia coli contains 70 to 80% PE and 20 to 25% PG (see Fig. S1 for structures). Within the differential lipids, all 4 lysophospholipids (lysoPLs; phospholipids with a single carbon chain) were highly accumulated in the *sepD* mutants. The distribution of PLs in the differential lipids was more complex. All 6 upregulated PLs were PEs, while out of the 10 PLs in the group of lipids that were downregulated in *sepD* mutants, 5 were PEs, 3 PGs, and 2 phosphatidylserines (PSs). All menaquinone and ubiquinone species were downregulated in the *sepD* mutants. Given these results, we focused our analyses on the PL and terpenoid-quinones pathways and identified lipids of these two main differential classes from the whole data set (Table S1).

10.1128/msystems.00202-22.7 TABLE S1 Identified lipids (taken from the lipidomics analysis of Δ*sepD* and WT/pCesT EPEC with respect to the wild type). PG, phosphatidyl glycerol; PE, phosphatidyl ethanolamide; PS, phosphatidyl serine; PA, phosphatidic acid; CL, cardiolipin. The lipid species were identified by MS^E^. The data shown represent the fold change calculated from the abundance of each lipid in the Δ*sepD* and WT/pCesT from the wild type. *, an intermediate ubiquinone. Download Table S1, XLSX file, 0.01 MB. Copyright © 2022 Zacharia et al. 2022 Zacharia et al. https://creativecommons.org/licenses/by/4.0/ This content is distributed under the terms of the Creative Commons Attribution 4.0 International license.

### T3SS activation drives a remodeling of the glycerophospholipid pathway.

**FIG 3:**
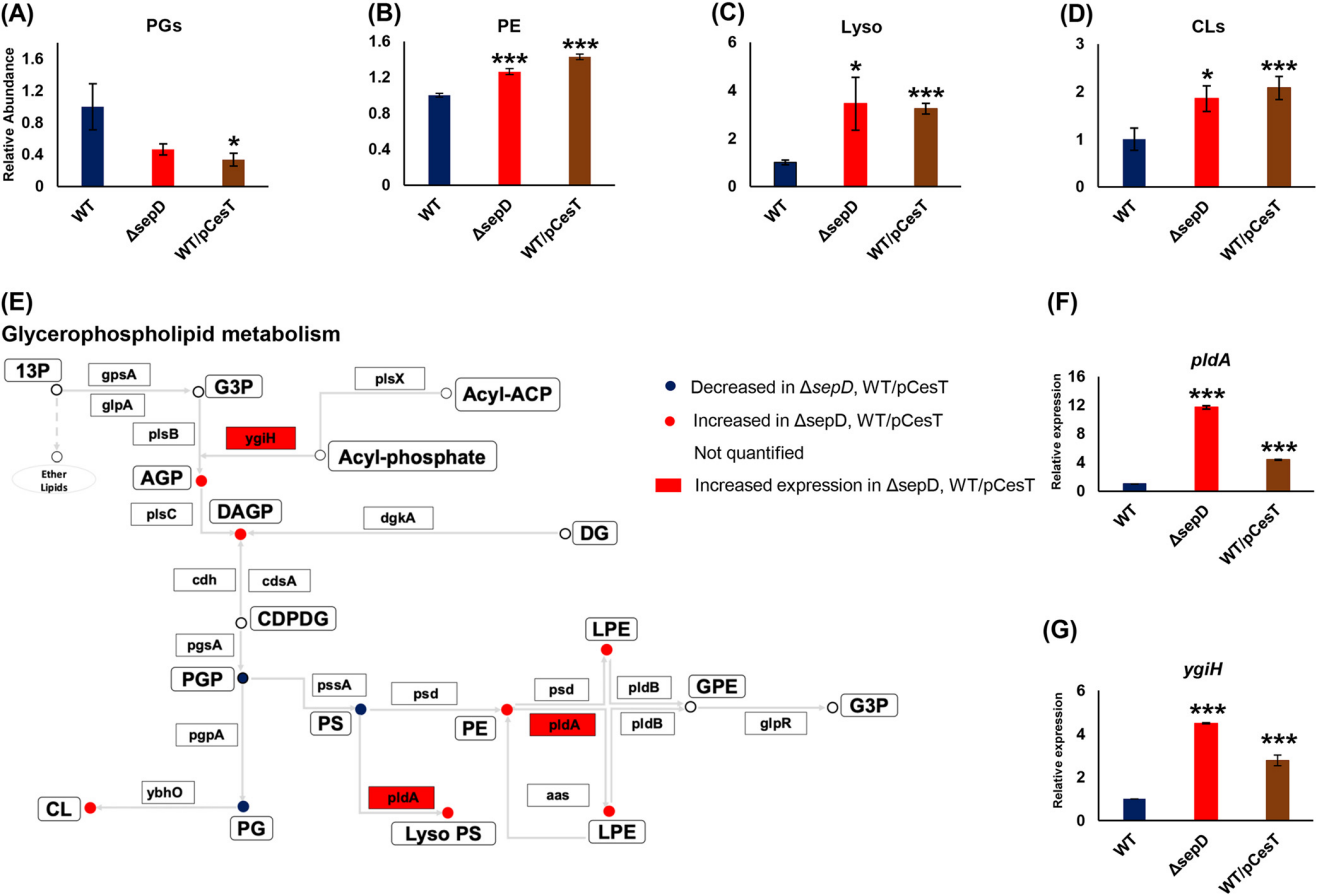
Repression of CsrA by CesT leads to a shift from phosphatidylglycerols (PGs) to phosphatidylethanolamines (PEs) and lysophospholipids (lysoPLs) on the one hand and cardiolipins (CLs) on the other. The relative total abundances of identified PGs (12 identified lipid species) (A), PEs (41 identified lipid species) (B), lysoPLs (34 identified lipid species) (C), and CLs (10 identified lipid species) (D) is presented. (E) A metabolic map of phospholipids was adapted from the Kyoto Encyclopedia of Genes and Genomes (KEGG) for Escherichia coli O127:H6 E2348/69 (EPEC) and modified. Lipid classes are represented by circles, with their names indicated. Lipid abbreviations: 13P, glycerone phosphate; G3P, glycerol-3-phosphate; AGP, acylglycerol-3-phosphate; DAGP, 1,2-diacylglycerol 3-phosphate; CDPDG, 1,2-diacylglycerol-cytidine-5-diphosphate; GPE, glycerophosphoethanolamine; LPE, lysophosphoethanolamine; Lyso PS, lysophosphoserine; PGP, phosphatidylglycerophosphate; PE, phosphatidylethanolamine; PS, phosphatidylserine; PG, phosphatidylglycerol. Enzymes are represented by arrows. Enzyme common nomenclature is used in the scheme. For an unambiguous identification, enzyme nomenclature (EC number system) is hereby given, along with further commonly used names: 1.1.1.94, GpsA, glycerol-3-phosphate dehydrogenase; 1.1.5.3, GlpD, *sn*-glycerol-3-phosphate dehydrogenase; 2.3.1.15, PlsB, glycerol-3-phosphate *O*-acyltransferase; 2.3.1.274, PlsX, phosphate acyltransferase; 2.3.1.275, PlsY (YgiH), glycerol-3-phosphate acyltransferase; 2.3.1.51, PlsC (ParF), 1-acyl-*sn*-glycerol-3-phosphate acyltransferase; 2.7.1.107, DgkA, diacylglycerol kinase; 3.6.1.26, Cdh, CDP-diacylglycerol phosphotidylhydrolase; 2.7.7.41, CdsA (Cds), CDP-diglyceride synthase; 2.7.8.5, PgsA, phosphatidylglycerophosphate synthetase; 3.1.3.27, PgpA (YajN), phosphatidylglycerophosphatase A; 2.7.8.8, PssA (Pss), phosphatidylserine synthase; 3.1.1.32, PldA, outer membrane phospholipase A; 4.1.1.65, Psd, phosphatidylserine decarboxylase; 3.1.1.4, PldA, outer membrane phospholipase A; 3.1.1.5, PldB, lysophospholipase L; and 3.1.4.46, GlpQ (UgpQ), glycerophosphodiester phosphodiesterase. (F and G) The relative expression of PldA and YgiH in the EPEC strains was quantified using quantitative PCR (qPCR); *n* = 4 for all data except for RT-qPCR, where *n* = 3. Data are presented as means ± SE. *, *P* < 0.05; ***, *P* < 0.001.

Our VIP analysis pointed to PLs and quinone terpenoids as major differential lipids classes following T3SS activation and implied a metabolic shift from PGs in the glycerophospholipid pathway. To better define the shifts in the pathway, we quantified the abundances of all identified phospholipid species per subclass, using our lipidomics analysis data. We identified 41 PE species and 12 PGs. We observed a shift in the PL composition. This shift was further verified using a second data set, where each PL subclass was quantified with a corresponding heavy isotope internal standard. All data presented here are for the latter quantified data set. We found a considerable decrease in the abundance of PGs in Δ*sepD* and WT/pCesT EPEC (Fig. 3A), whereas mild increase in that of PEs in WT/pCesT EPEC was observed (Fig. 3B). Aligned with our VIP analysis, we also revealed a higher abundance of the identified lysoPLs in Δ*sepD* and WT/pCesT EPEC (34 identified lipid species [Fig. 3C]). Cardiolipins (CLs; 10 identified lipid species [Fig. 3D]) were upregulated in the mutant strain as well. To pinpoint the enzymes involved in the shift in PL metabolism, and in particular the dramatic increase in the abundance of lysoPLs in the Δ*sepD* and WT/pCesT strains, we performed a network-based analysis of Δ*sepD* PL metabolism (Fig. 3E). This analysis underscored two enzymes: PldA, a phospholipase, and YgiH, which functions as a glycerol-3-phosphate acyltransferase for lysoPL biosynthesis (32). We then quantified the expression of the corresponding genes by quantitative reverse transcription-PCR (RT-qPCR) and noted a considerable upregulation in the RNA levels of these genes upon T3SS activation (Fig. 3F and G). These results are in alignment with the increased concentrations of the corresponding lipids.

Assuming that the changes we identified in PL metabolism may result in reduction of membrane integrity, we stained the membrane with the lipophilic dye FM4-64 and found a lower uptake of the dye in Δ*sepD* and CesT overexpression strains (Fig. S3).

10.1128/msystems.00202-22.3 FIG S3 Membrane stain analysis showing the lower uptake of membrane stain FM 4-64 in strains with high levels of CesT. Phase-contrast and fluorescent images of the EPEC wild type (WT) and mutants following FM 4-64 membrane staining. Scale bar indicates 5 μm (A). The fluorescence emissions from ~10,000 bacteria for each strain, in 3 technical replicates, were recorded, and the data are presented as means ± SE. ***, *P* < 0.001 (B). Download FIG S3, TIF file, 1.3 MB. Copyright © 2022 Zacharia et al. 2022 Zacharia et al. https://creativecommons.org/licenses/by/4.0/ This content is distributed under the terms of the Creative Commons Attribution 4.0 International license.

To assess the involvement of CsrA in the T3SS-dependent shift, we identified in PL metabolism, we first mined a *csrA* mutant transcriptome database for the glycerophospholipid pathway genes. We integrated our lipidomics data and the published gene expression (transcriptome sequencing [RNA-seq]) data for a better understanding of the PL network. The mined gene expression data were well aligned with the results of our analyses of the abundance of PL subclasses (Fig. S4A). To verify that the shift in PL metabolism was mediated by inhibition of CsrA by CesT, we quantified the PL subclasses in the EPEC Δ*csrA* mutant and compared them to those of the Δ*sepD* and wild-type strains. We found in the Δ*csrA* and Δ*sepD* mutants a similar shift from PGs to PEs and increased levels of LysoPLs (Fig. S4B to D). We then evaluated the expression of *pldA* and *ygiH* and could detect substantially higher expression in the Δ*csrA* and Δ*sepD* mutants than in wild-type EPEC (Fig. S4E to F). These results further suggest that the shift in PL metabolism was mediated via CsrA inhibition by CesT. The growth conditions in our study were set to mimic infection conditions (16, 24). Nevertheless, our gene expression analysis was in agreement with the mined data, although these data were obtained using EPEC grown under different conditions. Together, our results point to a CsrA-mediated regulation of PL metabolism in EPEC and suggest that this modulation is maintained under different growth conditions.

10.1128/msystems.00202-22.4 FIG S4 Data mining of *csrA* mutant transcriptome corroborated *csrA*-mediated regulation of the expression of phospholipid pathway genes by the T3SS. A metabolic map of phospholipids with data from our lipidomics analysis was integrated with transcriptomic data taken from Berndt et al. (V. Berndt, M. Beckstette, M. Volk, et al., Sci Rep 9:138, 2019, https://doi.org/10.1038/s41598-018-36932-w) (A). The glycerophospholipid pathway was adapted from the Kyoto Encyclopedia of Genes and Genomes (KEGG) for Escherichia coli O127:H6 E2348/69 (EPEC). Lipid classes are represented by circles, with their names indicated. Enzymes are represented by arrows, with their names indicated. Colors of circles (lipid classes) and quadrants (gene expression) are given as ln2 (fold change) relative to the wild-type mean value. Abbreviations used for lipid species names: 13P, glycerone phosphate; G3P, glycerol-3-phosphate; AGP, acylglycerol-3-phosphate; DAGP, 1,2-diacylglycerol 3-phosphate; CDPDG, 1,2-diacylglycerol-cytidine 5-diphosphate; GPE, glycerophosphoethanolamine; LPE, lysophosphoethanolamine; Lyso PS, lysophosphoserine; PGP, phosphatidylglycerophosphate; PE, phosphatidylethanolamine; PS, phosphatidylserine; PG, phosphatidylglycerol. Enzymes are represented by arrows, with their numbers indicated. Enzyme common nomenclature is used in the scheme. For an unambiguous identification, enzyme nomenclature (EC number system) is hereby given, along with further commonly used names: 1.1.1.94, GpsA, glycerol-3-phosphate dehydrogenase; 1.1.5.3, GlpD, *sn*-glycerol-3-phosphate dehydrogenase; 2.3.1.15, PlsB, glycerol-3-phosphate *O*-acyltransferase; 2.3.1.274, PlsX, phosphate acyltransferase; 2.3.1.275, PlsY (YgiH), glycerol-3-phosphate acyltransferase; 2.3.1.51, PlsC (ParF), 1-acyl-*sn*-glycerol-3-phosphate acyltransferase; 2.7.1.107, DgkA, diacylglycerol kinase; 3.6.1.26, Cdh, CDP-diacylglycerol phosphotidylhydrolase; 2.7.7.41, CdsA (Cds), CDP-diglyceride synthase; 2.7.8.5, PgsA, phosphatidylglycerophosphate synthetase; 3.1.3.27, PgpA (YajN), phosphatidylglycerophosphatase A; 2.7.8.8, PssA (Pss), phosphatidylserine synthase; 3.1.1.32, PldA, outer membrane phospholipase A; 4.1.1.65, Psd, phosphatidylserine decarboxylase; 3.1.1.4, PldA, outer membrane phospholipase A; 3.1.1.5, PldB, lysophospholipase L; and 3.1.4.46, GlpQ (UgpQ), glycerophosphodiester phosphodiesterase. The T3SS-related shift in the phospholipid composition is mediated by *csrA* (B to D). The total abundance of identified phospholipids—phosphatidylglycerols (PGs) (B), phosphatidylethanolamines (Pes) (C), and lysophospholipids (LysoPLs) (D)—is presented. (E and F) The expression of key enzymes responsible for the conversion of PLs to LysoPLs was evaluated by RT-qPCR: PldA (E) and YgiH (F). The data are presented as means ± SE (*n* = 4). **, *P* < 0.01; ***, *P* < 0.001. Download FIG S4, TIF file, 0.6 MB. Copyright © 2022 Zacharia et al. 2022 Zacharia et al. https://creativecommons.org/licenses/by/4.0/ This content is distributed under the terms of the Creative Commons Attribution 4.0 International license.

### Undecaprenyl lipid biosynthesis is upregulated, whereas synthesis of menaquinones and ubiquinones is downregulated, upon T3SS activation.

Isoprenoids (“terpenoids”) are structurally diverse lipids with skeletons composed of 5 carbon units. These units are formed from isopentenyl diphosphate (IPP) and dimethylallyl diphosphate (DMAPP) precursors and are successively assembled (33) (Fig. S1). All isoprenoids are synthesized by sequential head-to-tail 1′-4 condensations. Undecaprenyl phosphate is an essential long-chain isoprene lipid involved in the biogenesis of bacterial cell envelope carbohydrate polymers, including peptidoglycan and O antigen. It forms a membrane-bound carrier for the assembly of O-antigen units (34, 35). After synthesis, O antigen is ligated to the core lipid A, biosynthesizing lipopolysaccharide (LPS) (34). IPP is also the source of the prenyl side chains of the terpenoid quinones menaquinone and ubiquinone (36).

**FIG 4:**
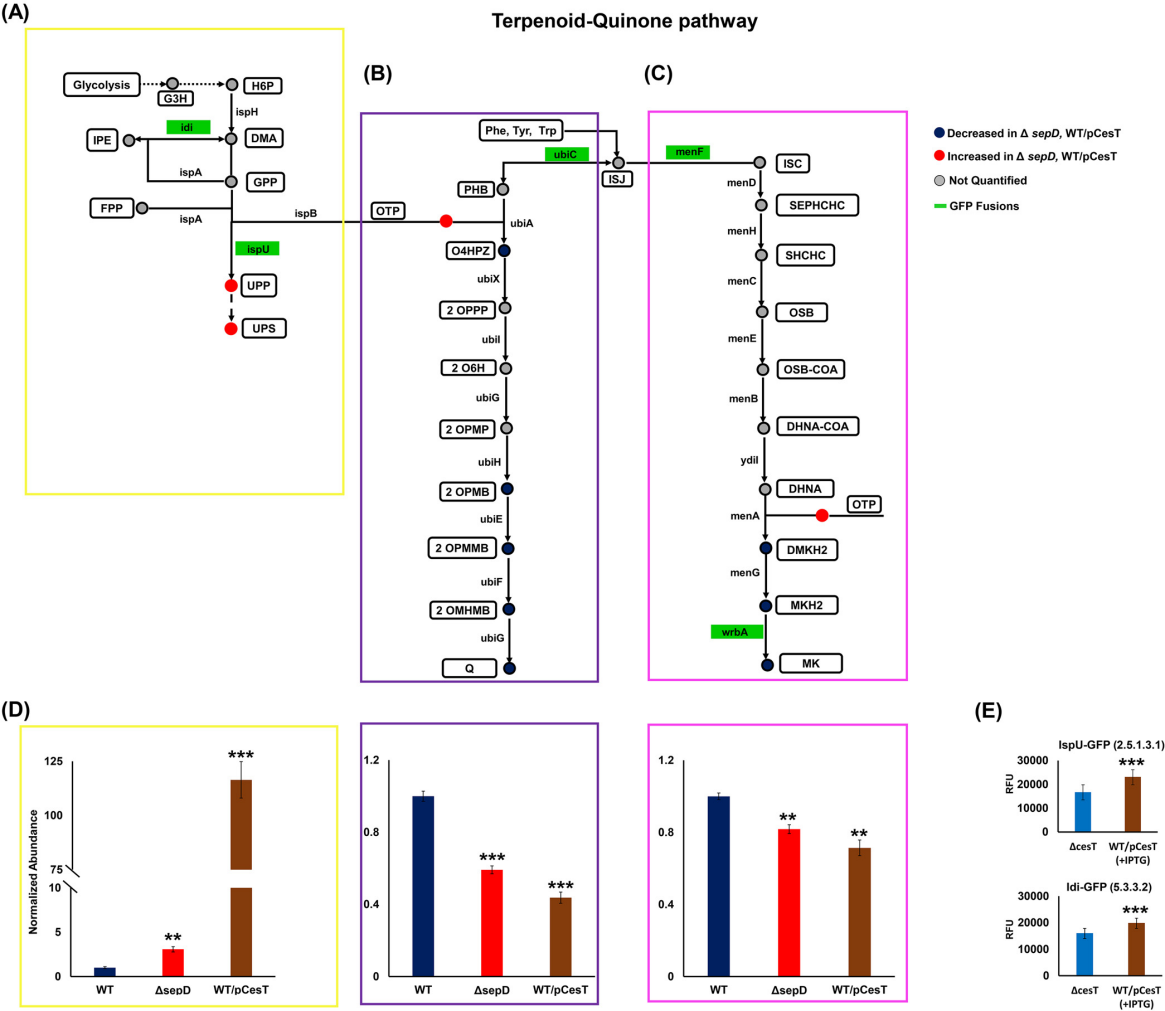
Mimicking the activation of T3SS in EPEC upregulates the biosynthesis of undecaprenyl lipids and downregulates the biosynthesis of menaquinones and ubiquinones. (A to C) The terpenoid quinone pathway was adapted from the KEGG for Escherichia coli O127:H6 E2348/69 (EPEC) and modified to include the undecaprenyl, ubiquinone, and menaquinone branches of the terpenoid pathway, resulting in a simplified scheme of the metabolic network of terpenoid quinone lipids. We divided the map into undecaprenyl (A; yellow box), ubiquinone (B; purple box), and menaquinone (C; pink box) lipids. The colors of lipid species (in circles) represent their relative accumulations according to our analyses. Lipid species and subclasses are represented in white boxes, and green boxes represent enzymes fused to GFP. Lipids abbreviations: H6P, 1-hydroxy-2-methyl-2-butenyl- 4-diphosphate; DMA, dimethylallyl diphosphate; IPE, isopentenyl diphosphate; GPP, geranyl diphosphate; FPP, farnesyl diphosphate; UPP, undecaprenyl diphosphate; UPS, undecaprenyl species; OTP, octaprenyldiphosphate; PHB, hydroxybenzoic acid; ISJ, chorismate; O4HPZ, 4-hydroxy-3-polyprenyl benzoate; 2OPPP, 2-octaprenyl phenol; 2O6H, 2-octaprenyl-6-hydroxyphenol; 2OPMP, 2-octaprenyl-6-methoxy phenol; 2OPMB, 2-octaprenyl-6-methoxy-1,4-benzoquinone; 2OPMMB, 2-octaprenyl-3-methyl-6-methoxy-1,4-benzoquinone; 2OMHMB, 2-octaprenyl-3-methyl-5-hydroxy-6-methoxy-1,4-benzoquinone; Q, ubiquinone; ISC, isochorismate; SEPHCHC, 2-succinyl-5-enolpyruvyl-6-hydroxy-3-cyclohexene-1-carboxylate; SHCHC, 6-hydroxy-2-succinylcyclohexa-2,4-diene-1-carboxylate; OSB, 2-succinylbenzoate; OSB-COA, 2-succinylbenzoyl coenzyme A (CoA); DHNA-CoA, 1,4-dihydroxy-2-naphthoyl-CoA; DHNA, 1,4-dihydroxy-2-naphthoate; DMKH2, demethylmenaquinol; MKH2, menaquinol; MK, menaquinone. Enzymes are represented by arrows, with their names indicated. Enzyme common nomenclature is used in the scheme. For an unambiguous identification, enzyme nomenclature (EC number system) is hereby given, along with further commonly used names: 1.17.7.4, IspH (YaaE, LytB), 4-hydroxy-3-methylbut-2-enyl diphosphate reductase; 2.5.1.1, 2.5.1.10, IspA, farnesyl diphosphate synthase; 2.5.1.90, IspB (Cel, YhbD), all-trans-octaprenyl-diphosphate synthase; 2.5.1.31, IspU (UppS, Rth, YaeS), ditrans, polycis-undecaprenyl-diphosphate synthase; 4.1.3.40, UbiC, chorismate lyase; 2.5.1.39, UbiA, 4-hydroxybenzoate octaprenyltransferase; 2.5.1.129, UbiX (DedF), 3-octaprenyl-4-hydroxybenzoate carboxy-lyase; 1.14.13.240, UbiI (VisC), 2-methoxy-6-(all-trans-octaprenyl)phenol 4-hydroxylase; 2.1.1.222, 2.1.1.64, UbiG (PufX, YfaB), bifunctional 3-demethylubiquinone-8 3-*O*-methyltransferase and 2-octaprenyl-6-hydroxyphenolmethylase; 1.14.13.-, UbiH (Acd, VisB), 2-octaprenyl-6-methoxyphenol 4-hydroxylase; 2.1.1.201, UbiE (YigO), bifunctional 2-octaprenyl-6-methoxy-1,4-benzoquinol methylase and demethylmenaquinone methyltransferase; 1.14.99.60, UbiF (YleB), 2-octaprenyl-3-methyl-6-methoxy-1,4-benzoquinol oxygenase, 2-octaprenyl-3-methyl-6-methoxy-1,4-benzoquinol hydroxylase; 5.4.4.2, MenF (YfbA), isochorismate synthase 2; 2.2.1.9, MenD, 2-succinyl-5-enolpyruvyl-6-hydroxy-3-cyclohexene-1-carboxylatesynthase; 4.2.99.20, MenH (YfbB), 2-succinyl-6-hydroxy-2,4-cyclohexadiene-1-carboxylate synthase; 4.2.1.113, MenC, *o*-succinylbenzoate synthase; 6.2.1.26, MenE, *o*-succinylbenzoate CoA ligase; 4.1.3.36, MenB, 1,4-dihydroxy-2-naphthoyl-CoA synthase; 3.1.2.28, YdiI (MenI), 1,4-dihydroxy-2-naphthoyl-CoA hydrolase; 2.5.1.74, MenA (YiiW), 1,4-dihydroxy-2-naphthoate octaprenyltransferase; 2.1.1.163, 2.1.1.201, UbiE (MenG, YigO), demethylmenaquinone methyltransferase/2-methoxy-6-octaprenyl-1,4-benzoquinol methylase; and 1.6.5.2, WrbA (YtfG), quinone oxidoreductase. (D) Accumulated abundances of undecaprenyl, ubiquinone, and menaquinone lipids following accumulation of CesT in the bacteria. (E) GFP fluorescence was measured in the strains with null CesT and WT/pCesT. Their expression was determined in terms of relative fluorescence units. Data are presented as means ± SEM (*n* = 4). **, *P* < 0.01; ***, *P* < 0.001.

Our lipidomics analyses suggested that quinone terpenoid biosynthesis is altered in *sepD* mutants (Fig. 2C). We thus used the data set generated by this lipidomics analysis to compare the levels of identified isoprenoid species per subclass. This analysis pointed to increased accumulation of undecaprenyl end products in the Δ*sepD* mutant compared to those of a wild-type strain, but decreased ubiquinone and menaquinone end product accumulation, suggesting a shift toward undecaprenyl biosynthesis in the *sepD* mutants (Fig. 4A to D). This shift was recapitulated and was, in fact, more pronounced in EPEC overexpressing CesT (Fig. 4A to D).

We then tested the influence of CesT levels on the production of key enzymes involved in the terpenoid quinone biosynthesis. To this end, we fused a green fluorescent protein (GFP) reporter gene in frame to the 3′ ends of a selected number of genes in their native chromosomal location without perturbing their transcriptional unit. Thus, the GFP levels provide a means to measure enzyme production. We selected for tagging genes of which *gfp* tagging was previously found to be tolerated by the bacteria (37). Using this approach, we *gfp* tagged the following genes: *idi*, which forms isopentenyl pyrophosphate, an isoprenoid precursor; *ispU*, involved in the biosynthesis of undecaprenyl pyrophosphate; *ubiC* and *menF*, bridging between menaquinone and ubiquinone pathways; and *wrbA*, encoding menaquinone biosynthesis. To enhance the signal/noise ratio, we constructed the GFP reporters in WT/pCesT and Δ*cesT* strains grown statically in DMEM at 37°C. We detected upregulation of Idi-GFP and IspU-GFP levels upon *cesT* overexpression (Fig. 4E). This increase is in agreement with our lipidomics analyses. Changes in the levels of GFP-tagged UbiC, MenF, and WrbA, involved in menaquinone and ubiquinone production, were not significant (Fig. S5).

10.1128/msystems.00202-22.5 FIG S5 No change noted in the expression of UbiC, MenF, and WrbA following CesT accumulation. Quinone terpenoid biosynthesis genes were GFP labeled in EPEC null for CesT or overexpressing it. GFP fluorescence intensity was then measured. Data are presented as means ± SE (*n* = **3**). Download FIG S5, TIF file, 0.1 MB. Copyright © 2022 Zacharia et al. 2022 Zacharia et al. https://creativecommons.org/licenses/by/4.0/ This content is distributed under the terms of the Creative Commons Attribution 4.0 International license.

Our lipidomics analyses and the quantification of the expression of *gfp*-tagged *idi* and *ispU* are well aligned with the data we mined on the expression of genes of the quinone terpenoid pathways in *ΔcsrA* EPEC (Fig. S6).

10.1128/msystems.00202-22.6 FIG S6 Data mining in the Δ*csrA* transcriptome (V. Berndt, M. Beckstette, M. Volk, et al., Sci Rep 9:138, 2019, https://doi.org/10.1038/s41598-018-36932-w) suggests upregulation of undecaprenyl biosynthesis. The network was adapted from Kyoto Encyclopedia of Genes and Genomes (KEGG) for Escherichia coli O127:H6 E2348/69 (EPEC) and modified to include the undecaprenyl, ubiquinone, and menaquinone branches of the terpenoid pathway. Lipids are represented by circles, with their names indicated. Enzyme are represented by arrows, with their names indicated. The colors represent the log_2_ fold change between the *csrA* mutant and the WT strain. Lipid abbreviations: H6P, 1-hydroxy-2-methyl-2-butenyl- 4-diphosphate; DMA, dimethylallyl diphosphate; IPE, isopentenyl diphosphate; GPP, geranyl diphosphate; FPP, farnesyl diphosphate; UPP, undecaprenyl diphosphate; OTP, octaprenyldiphosphate; PHB, hydroxybenzoic acid; ISJ, chorismate; O4HPZ, 4-hydroxy-3-polyprenyl benzoate; 2OPPP, 2-octaprenyl phenol; 2O6H, 2-octaprenyl-6-hydroxyphenol; 2OPMP, 2-octaprenyl-6-methoxy phenol; 2OPMB, 2-octaprenyl-6-methoxy-1,4-benzoquinone; 2OPMMB, 2-octaprenyl-3-methyl-6-methoxy-1,4-benzoquinone; 2OMHMB, 2-octaprenyl-3-methyl-5-hydroxy-6-methoxy-1,4-benzoquinone; Q, ubiquinone; ISC, isochorismate; SEPHCHC, 2-succinyl-5-enolpyruvyl-6-hydroxy-3-cyclohexene-1-carboxylate; SHCHC, 6-hydroxy-2-succinylcyclohexa-2,4-diene-1-carboxylate; OSB, 2-succinylbenzoate; OSB-COA, 2-succinylbenzoyl coenzyme A (CoA); DHNA-CoA, 1,4-dDihydroxy-2-naphthoyl-CoA; DHNA, 1,4-dihydroxy-2-naphthoate; DMKH2, demethylmenaquinol; MKH2, menaquinol; MK, menaquinone. Enzyme common nomenclature is used in the scheme. For an unambiguous identification, enzyme nomenclature (EC number system) is hereby given, along with further commonly used names: 1.17.7.4, IspH (YaaE, LytB), 4-hydroxy-3-methylbut-2-enyl diphosphate reductase; 2.5.1.1, 2.5.1.10, IspA, farnesyl diphosphate synthase; 2.5.1.90, IspB (Cel, YhbD), all-trans-octaprenyl-diphosphate synthase; 2.5.1.31, IspU (UppS, Rth, YaeS), ditrans, polycis-undecaprenyl-diphosphate synthase; 4.1.3.40, UbiC, chorismate lyase; 2.5.1.39, UbiA, 4-hydroxybenzoate octaprenyltransferase; 2.5.1.129, UbiX (DedF), 3-octaprenyl-4-hydroxybenzoate carboxy-lyase; 1.14.13.240, UbiI (VisC), 2-methoxy-6-(all-trans-octaprenyl)phenol 4-hydroxylase; 2.1.1.222, 2.1.1.64, UbiG (PufX, YfaB), bifunctional 3-demethylubiquinone-8 3-*O*-methyltransferase and 2-octaprenyl-6-hydroxyphenolmethylase; 1.14.13.-, UbiH (Acd, VisB), 2-octaprenyl-6-methoxyphenol 4-hydroxylase; 2.1.1.201, UbiE (YigO), bifunctional 2-octaprenyl-6-methoxy-1,4-benzoquinol methylase and demethylmenaquinone methyltransferase; 1.14.99.60, UbiF (YleB), 2-octaprenyl-3-methyl-6-methoxy-1,4-benzoquinol oxygenase, 2-octaprenyl-3-methyl-6-methoxy-1,4-benzoquinol hydroxylase; 5.4.4.2, MenF (YfbA), isochorismate synthase 2; 2.2.1.9, MenD, 2-succinyl-5-enolpyruvyl-6-hydroxy-3-cyclohexene-1-carboxylatesynthase; 4.2.99.20, MenH (YfbB), 2-succinyl-6-hydroxy-2,4-cyclohexadiene-1-carboxylate synthase; 4.2.1.113, MenC, *o*-succinylbenzoate synthase; 6.2.1.26, MenE, *o*-succinylbenzoate CoA ligase; 4.1.3.36, MenB, 1,4-dihydroxy-2-naphthoyl-CoA synthase; 3.1.2.28, YdiI (MenI), 1,4-dihydroxy-2-naphthoyl-CoA hydrolase; 2.5.1.74, MenA (YiiW), 1,4-dihydroxy-2-naphthoate octaprenyltransferase; 2.1.1.163, 2.1.1.201, UbiE (MenG, YigO), demethylmenaquinone methyltransferase/2-methoxy-6-octaprenyl-1,4-benzoquinol methylase; and 1.6.5.2, WrbA (YtfG), quinone oxidoreductase. Download FIG S6, TIF file, 1.0 MB. Copyright © 2022 Zacharia et al. 2022 Zacharia et al. https://creativecommons.org/licenses/by/4.0/ This content is distributed under the terms of the Creative Commons Attribution 4.0 International license.

**FIG 5:**
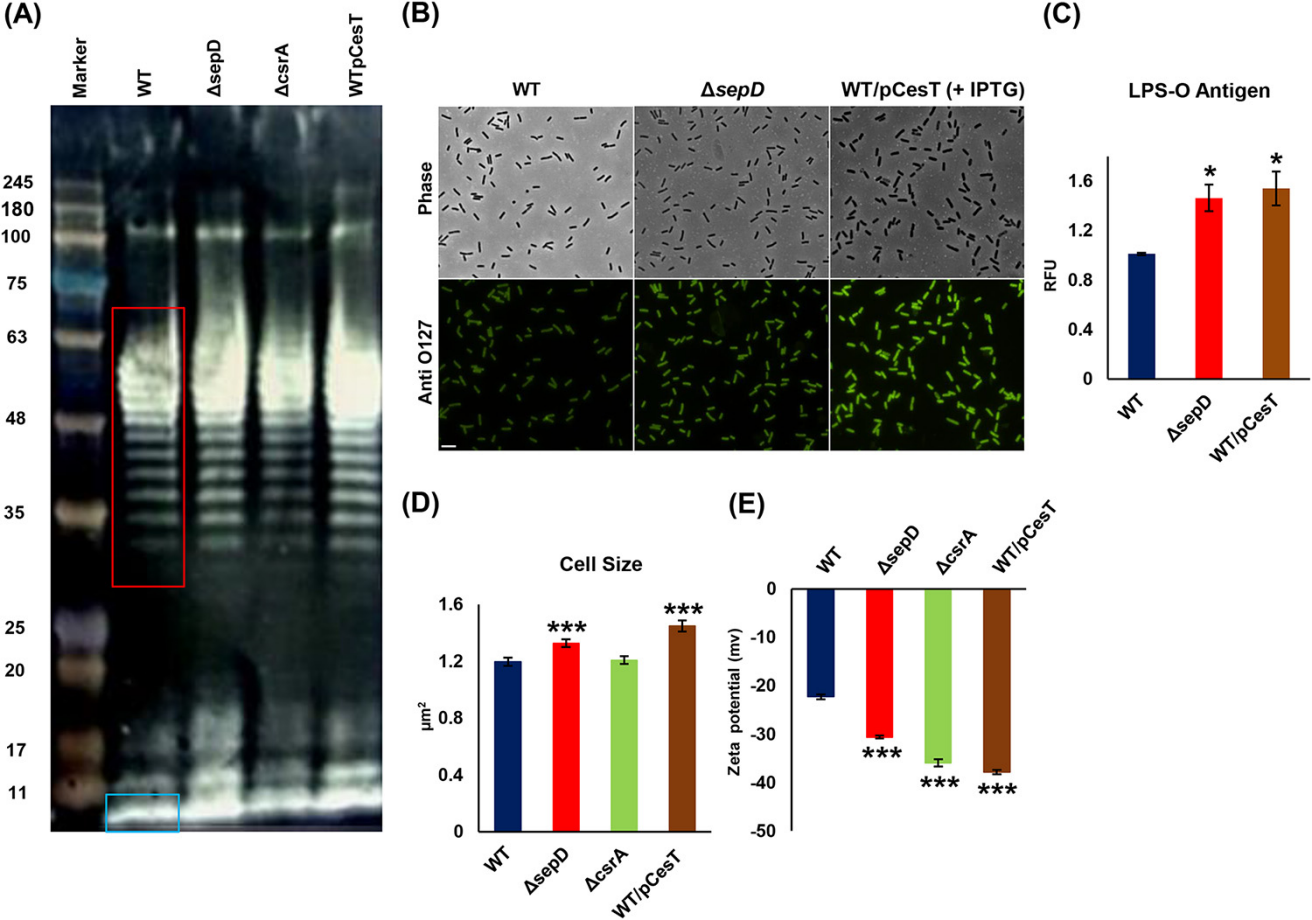
O-antigen levels, cell size, and charge potential in EPEC increase upon T3SS activation. EPEC strains were subcultured in DMEM at 37°C to an OD_600_ of 0.6. (A) LPS was extracted and subjected to Western blotting. O-antigen repeats are boxed in red, and the core is in a blue box. (B and C) Bacteria were fixed, incubated with anti-O127 antibody, and visualized under phase-contrast and fluorescence microscopy. Scale bar indicates 5 μm. The fluorescence intensity was measured using NIS elements AR 4.3, and the relative fluorescence was determined. The data are presented as means ± SEM (*n* = 3). (D and E) EPEC strains were subcultured in DMEM at 37°C to an OD_600_ of 0.6. Bacterial cell size (*n* = 150) (D) was measured under a microscope using ImageJ software. (E) Zeta potential was measured using Zetasizer Nano ZS. Zeta potential measurements for each sample and the mean charge were recorded (*n* = 12). Data are presented as means ± SEM (*n* = 3). *, *P* < 0.05; ***, *P* < 0.001.

Taken together, our analyses point to a metabolic shift toward undecaprenyl species upon T3SS activation. Importantly, undecaprenyl pyrophosphate (UPP) is required for the synthesis of O-antigen repeating units on surface of the inner leaflet of the inner membrane and for the flipping of the UPP–O-antigen complex to the outer leaflet, where it is used as a precursor for the biosynthesis of the LPS O antigen and O-antigen capsule (38). We assumed that the increase in the level of undecaprenyl species we monitored in *sepD* mutants would be reflected in higher biogenesis of O antigen. To define possible alterations in O-antigen levels, we carried out Western blot analysis of O-antigen repeats, which suggested increased levels of LPS O antigen in the Δ*sepD* mutant and *cesT*-overexpressing EPEC strains (Fig. 5A). Immunofluorescence microscopy demonstrated increased levels of O antigen (LPS and/or capsular) on the surface of the Δ*sepD* mutant and *cesT*-overexpressing EPEC strains (Fig. 5B and C), in concordance with the total O-antigen levels observed in Western blot analysis.

We reasoned that the changes we found in the composition of EPEC membrane lipids should be reflected in the physiological and morphological characteristics, and we measured the cell size of the Δ*sepD* mutant and WT/pCesT. We found significant increases in their sizes (Fig. 5D), consistent with previous observations made for *csrA* mutant E. coli (23). Surprisingly, however, the size of the *csrA* mutant was similar to that of wild-type EPEC in our study. The O-antigen content and composition may influence cell surface charge, which plays a role in adhesion of bacteria to surfaces. We saw an increase in the negative charge of the T3SS-activated strains (Fig. 5E).

**FIG 6:**
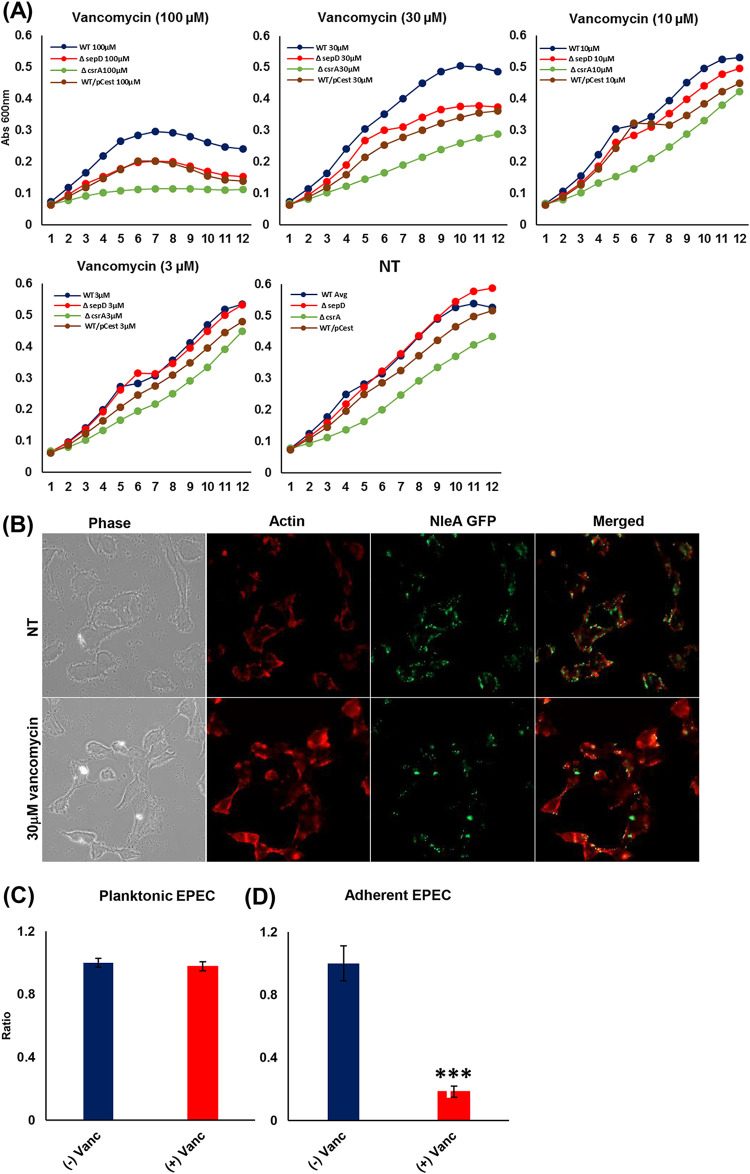
T3SS activation upon attachment to host cells increases the sensitivity of EPEC to vancomycin. (A) The sensitivity of EPEC strains was tested in T3SS-activated EPEC (Δ*sepD* and WT/pCesT) versus the WT by a vancomycin resistance assay, where the growth curve was monitored at OD_600_ throughout 12 h. The vancomycin sensitivity of T3SS-activated strains versus the WT at various concentrations (3, 10, 30, and 100 μM) and its respective nontreated (NT) control are shown (A). To confirm the CsrA-mediated response to T3SS activation, we also tested the vancomycin sensitivity of *csrA* mutants. The data are presented as means ± SEM (*n* = 3). The NleA-GFP EPEC strain, which exhibits fluorescence upon attachment to the host cells, was examined microscopically. NleA-GFP EPEC cells were grown in high-glucose DMEM in the presence or absence (NT) of vancomycin (30 μM) (nontoxic for planktonic EPEC) for 4.5 h in a 24-well plate. The cells were fixed and stained with rhodamine phalloidin. The infection was visualized using an Axio Observer Z1 microscope, and the image was processed with Zen pro-2012 (B). Planktonic EPEC treated with vancomycin (30 μM) showed no effect on their growth (C). To study the effect of vancomycin on adherent cells, HeLa cells were infected with wild-type EPEC in the presence of vancomycin (30 μM). After 3 h of infection, attached EPEC organisms were recovered and plated onto LB agar. Following overnight incubation at 37°C, colonies were counted, and the ratio of vancomycin treated to nontreated organisms was assessed (D). The data shown are averages from 3 experiments performed (*n* = 6 in each experiment) ± SEM. ***, *P* < 0.001.

The outer and inner membranes form a barrier that separates and protects the bacteria from the extracellular environment. Given the profound changes we observed in the lipid composition of the EPEC membranes upon activation of the T3SS, we sought to study the influence of the T3SS on membrane permeability by using a vancomycin permeability-mediated resistance assay (39). We detected higher sensitivity to vancomycin in Δ*sepD* and WT/pCesT EPEC than in the wild-type strain (Fig. 6A). To confirm the physiological relevance of the changes in membrane permeability, we wished to evaluate the permeability of adherent versus planktonic wild-type EPEC. For this aim, we monitored adherent NleA-GFP EPEC (which exhibit fluorescence upon attachment to the host cells) in coculture with HeLa cells. Our microscopic examination suggested a decrease in the numbers of adherent EPEC cells upon treatment with 30 μM of vancomycin (nontoxic for planktonic EPEC) (Fig. 6B). Vancomycin treatment also resulted in lower pedestal formation (Fig. 6B). Importantly, no effect of vancomycin on planktonic EPEC bacteria could be detected (Fig. 6C). To test the influence of vancomycin on adherent EPEC, we infected HeLa cells with wild-type EPEC and treated them with vancomycin (30 μM). After infection, adherent EPEC were detached from the host using Triton X-100 and plated for CFU determination. The data show that attached EPEC became much more sensitive to vancomycin than nonattached EPEC (Fig. 6D).

**FIG 7:**
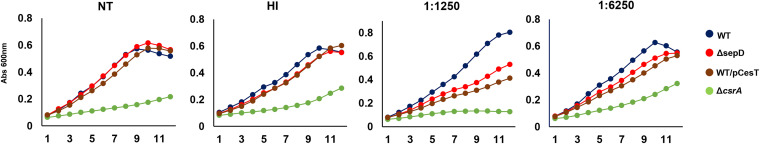
Following T3SS activation, EPEC organisms exhibit lower resistance to serum bactericidal components. Wild-type, Δ*sepD*, Δ*csrA*, and WT/pCesT strains were grown overnight in media containing human sera of different dilutions, no serum (NT), or heat-inactivated sera (HI) at 37°C in a Spark 10M microplate reader. Bacterial growth kinetics was monitored at hourly intervals for 12 h. The data represented are mean values of 3 replicates.

To further define the influence of T3SS on the interactions between the host and bacterial membrane, we cultured the bacterial strains with diluted serum, and we noted a reduced resistance to serum in the Δ*sepD* and WT/pCesT mutants (Fig. 7).

## DISCUSSION

In this study, we defined a modulation of membrane lipid composition of pathogenic E. coli by a secretion system—the T3SS—and demonstrated its implications on membrane function.

An important motivation for carrying out this study was elucidating how attachment to the host and the activation of the T3SS would influence EPEC membrane composition and metabolism.

We also expanded the scope of the metabolic regulation by CsrA. Many studies demonstrate the impact of CsrA on E. coli central carbon metabolism and physiology, mostly in the laboratory K-12 model strains (22, 25–28) but also in other E. coli isolates (40), including EPEC (30). Our study elucidates a new aspect of the CsrA function that was previously overlooked. We show here the critical impact of T3SS/CesT regulation of CsrA in controlling E. coli lipid metabolism and membrane function. Linking the changes we found in lipid metabolism to changes in other metabolic networks, and in particular to central carbon metabolism, would be of interest. Importantly, our results point to the dependency of EPEC lipid metabolism on host attachment and associate it with CsrA repression, most notably, a shift towards lysoPLs and a shift from menaquinones and ubiquinones toward the production of undecaprenyl lipids. We consider that Δ*csrA* mutant EPEC would only partially phenocopy the physiological state of attached EPEC. This is because this mutant shows reduced growth rate and, in addition, may collect suppressor mutations. To better mimic T3SS activation upon host attachment, we capitalized on engineered EPEC strains that transiently and partially inhibit CsrA (as in attached EPEC) and show a growth rate similar to that of the wild-type EPEC under our experimental conditions. Using these strains, we carried out a lipidomics study. Altogether, our data are in agreement with available transcript databases of *csrA* mutant EPEC. In a previous analysis by Berndt and colleagues, EPEC organisms were cultivated on modified M9 medium supplemented with low glucose (0.02 to 0.2% [wt/vol] glucose; incubated at 37°C and 160 rpm), and their metabolomes were examined (30). Our lipidomics analyses point to higher levels of lysoPLs in T3SS-activated EPEC, whereas the data of Berndt et al. disagree with this premise. This discrepancy may reflect differences in experimental conditions used in the two studies. For example, we grew the bacteria statically in DMEM to an OD_600_ of 0.6, while Berndt et al. used modified M9 medium and grew the bacteria to an OD_600_ of 1.0. Notably, the critical factor in selecting our growth conditions was mimicking infection conditions by keeping them static. The seeming discrepancy could also be related to differences in the methods used for sample preparation. We used a lipid extraction system, whereas Berndt and colleagues used an extraction system optimized for species with medium polarity. Further to the PL remodeling, our unbiased lipidomics analysis suggested alterations in the metabolism of terpenoid quinones. To better understand the changes in EPEC metabolism following T3SS activation, we mined a database of gene expression alterations in *csrA* mutant EPEC (30). The data mined implied an upregulation of the undecaprenyl branch of the terpenoid quinone pathway in the *csrA* mutant (Fig. S6). Whether and how the observed shifts in lipid metabolism contribute to host colonization are key questions that remain to be addressed in future studies. Moreover, following up on our findings in bacteria *in vivo* will be important to establish their possible clinical implications. One possible mechanism to such potential contribution is the regulation of zeta potential, reflecting the degree of electrostatic repulsion. Our finding of higher zeta potentials in the strains that mimic a postattachment condition may imply that bacterial cells remodel the membrane charge to avert membranous fusion with the host cells.

Bacteria use terpenoids like undecaprenyl phosphate as lipid carriers to assemble numerous glycan polymers that comprise the bacterial envelope. In E. coli, undecaprenyl phosphate is required for synthesizing peptidoglycan, O antigen, and exopolysaccharides (41). Importantly, *idi* removal suppressed a mutation in undecaprenyl pyrophosphate synthase (important for synthesizing undecaprenyl phosphate) (42). Accordingly, our findings that the expression of both *idi* and *ispU* is higher in the *sepD* mutant and in the CesT-overexpressing strains suggest that EPEC attachment to the host cell may remodel its envelope structure. This notion is supported by the considerable modulation of O-antigen content in the envelope of *sepD* and the CesT-overexpressing strains. The regulation of envelope structure by the T3SS results in an increase in membrane permeability upon activation of the T3SS, evident from the higher sensitivity of T3SS mutants to large-scaffold antibiotics such as vancomycin. The high sensitivity of adherent wild-type EPEC to vancomycin underscores the physiological implications of this finding. The dramatic reduction in the numbers of attached EPEC organisms due to concentrations (nontoxic for planktonic EPEC) of vancomycin may also be the result of lower adherence in subtoxic concentrations of vancomycin.

Our lipidomics analyses that point to a considerable increase in the abundance of lysoPLs under conditions that mimic EPEC attachment are of interest. LysoPLs produced by host intestinal cells induce a cAMP-dependent signaling pathway in infecting Salmonella, resulting in the production and secretion of active flagellin (43). Thus, it would be intriguing to test if bacteria might use self-produced lysoPLs as a second messenger. It is possible that lysoPL production and secretion may contribute to the capacity of EPEC to evade the host immune response.

In conclusion, our work provides a comprehensive account of the T3SS-dependent lipid metabolism and membrane biogenesis via CsrA repression in EPEC. It provides the foundation for the study of regulation of lipid metabolism and membrane function by bacterial secretion systems. Our data provide a first insight into the remodeling of bacterial membrane lipid composition upon host attachment, suggesting a metabolic switch from the planktonic to the cell-adherent lifestyle.

## MATERIALS AND METHODS

### Bacterial cultures.

Bacterial strains and plasmids used are presented in Table S2 and primers in Table S3. All bacterial strains are isogenic. Bacteria were grown in Luria-Bertani (LB) broth supplemented, when needed for subculture, with ampicillin (100 μg/mL), streptomycin (50 μg/mL), chloramphenicol (25 μg/mL), kanamycin (30 μg/mL), or tetracycline (10 μg/mL). For lipid extraction, EPEC strains were grown statically at 37°C overnight in LB medium with respective antibiotics. For all experiments, overnight-grown bacterial cultures were then diluted 1:50 using high-glucose Dulbecco’s modified Eagle medium (DMEM; Biological Industries) lacking pyruvate and glutamate and statically grown at 37°C. They were kept under these growth conditions until reaching an OD_600_ of 0.6.

10.1128/msystems.00202-22.8 TABLE S2 Strains and plasmids used for cloning. Download Table S2, XLSX file, 0.01 MB. Copyright © 2022 Zacharia et al. 2022 Zacharia et al. https://creativecommons.org/licenses/by/4.0/ This content is distributed under the terms of the Creative Commons Attribution 4.0 International license.

10.1128/msystems.00202-22.9 TABLE S3 Primer sequence for GFP fusions in EPEC. Download Table S3, XLSX file, 0.01 MB. Copyright © 2022 Zacharia et al. 2022 Zacharia et al. https://creativecommons.org/licenses/by/4.0/ This content is distributed under the terms of the Creative Commons Attribution 4.0 International license.

To express CesT in strains containing the pCesT plasmid, 0.05 mM IPTG was added 3 h after culturing in DMEM. Bacterial growth was extended to an OD_600_ of 0.6.

### Cloning, GFP fusion, and bacterial strain construction.

For GFP fusion, bacteria were electroporated with pKD46 plasmid harboring λ Red genes (γ, β, and Exo) (44). The *tet-sacB* cassette (45) was introduced downstream to the desired gene of interest and replaced with GFP insertion. GFP fusions were verified using PCR with flanking primers. Primers for PCR confirmation of GFP fusions are shown in Table S3. For overexpression of CesT levels, the pKD46 plasmids were cured, and pSA10 plasmid containing *CesT* was electroporated. Overexpression of CesT was induced with IPTG.

### RNA extraction and reverse transcription-PCR.

Overnight-grown EPEC organisms were subcultured in high-glucose DMEM. Following centrifugation, cell pellets were reconstituted in Tris-acetate-EDTA (TAE) buffer and three freeze-thaw cycles were performed. Total RNA was extracted (Direct-zol RNA miniprep kit; Zymo), and 1.5 μg was treated with RQ1 DNase I (Promega; 1 U/μg of RNA). cDNA was synthesized with a qPCRBIO high-quality cDNA synthesis kit and quantified by real-time PCR using a SYBR green mix (Absolute SYBR green ROX mix; Thermo). 16S rRNA (*rrsB*) was used as a housekeeping gene. Primers for qRT-PCR are shown in Table S3.

### Solvents and reagents for lipidomics analysis.

Acetonitrile, methanol (both ultra-liquid chromatography-mass spectrometry [ultra-LC-MS] grade), chloroform, and water (high-performance liquid chromatography [HPLC]-MS grade) were supplied by J.T. Baker, isopropanol (HPLC-MS grade) was from ChemSolute, and formic acid (HPLC-MS grade) was supplied by TCI. Ammonium fluoride (>99%) was supplied by Sigma-Aldrich. Internal standard mix, EquiSPLASH LIPIDOMIX (MS-quantitative grade), was obtained from Avanti Polar Lipids.

### Sample preparation for lipidomics analyses.

Cells were centrifuged (5,000 × *g* for 10 min at 4°C) and washed twice with 20 mL of cold phosphate-buffered saline (PBS), and bacterial pellets (45 mg, wet weight) were immediately snap-frozen in liquid nitrogen, stored for overnight at −80°C, and analyzed the following day. Bacteria (45 mg, wet pellet) were reconstituted in 400 μL of LC-MS grade water and transferred to glass tubes. Following the addition of 800 μL of ice-cold methanol and internal standards (EquiSPLASH LIPIDOMIX), samples went through five cycles of 30-s ultrasonication at 4°C for quenching and complete lysis. A total of 400 μL of cold chloroform was added, and another sequence of 30 s × 5 sonication cycles at 4°C was carried out. The tubes were incubated at room temperature for 30 min with occasional mixing (46) and then centrifuged at 770 × *g* for 10 min at 4°C for phase separation. The lower chloroform phase was transferred to clean glass tubes, and the protein disk at interface was reextracted with the same solvent system. Protein disk extracts were then pooled with the respective sample. Samples were concentrated in vacuum concentrator (126 SC210A SpeedVac; Thermo Scientific) and reconstituted in 200 μL of 95% acetonitrile and 0.1% Formic Acid (FA). Samples were filtered through Acrodisc polytetrafluoroethylene (PTFE) membrane filters (0.2 μm; Pall Corporation, USA) and transferred to Waters ACQUITY ultraperformance liquid chromatography (UPLC) 700-μL round 96-well sample plate.

### UPLC-MS analysis.

LC-MS analysis was carried out in a Waters Acquity UPLC H-class (Waters, Milford, MA) and Xevo G2-XS high-resolution, high-mass-accuracy quadrupole time of flight (Q-TOF) MS (Waters, Manchester, UK) system. LC-MS runs for lipidomics analyses were performed using a UPLC CSH C_18_ column (2.1×100 mm, 1.7 μm; Waters). The column temperature was maintained at 60°C. The mobile phase consisted of 0.1% (vol/vol) FA in water (solvent A), 0.1% FA in acetonitrile (solvent B), and isopropanol (solvent C). A flow rate of 0.4 mL/min was used with a linear gradient (Table S4). Electrospray ionization (ESI)-MS was calibrated using sodium formate, and leucine enkephalin was used as the lock mass (*m/z* 556.2771, 200 pg/mL) and continuously infused at 6 μL/min. The capillary spray was maintained at 3.0 kV; the data were acquired in positive and negative modes with collision energies of 15 to 45 eV and 30 to 60 eV, respectively. Full‐scan and MS^E^ data acquisition were performed, ranging from 30 to 2,000 Da. Argon was used as the collision gas for collision‐induced dissociation. Ammonium fluoride was used for postcolumn derivatization to improve the yields of the neutral charged lipids in the ES+ mode. MassLynx 4.1 (Waters Corporation, Milford, MA) was used to control the instrument, calculate accurate masses, and for mass spectral visualization.

10.1128/msystems.00202-22.10 TABLE S4 The linear gradient for the lipidomics analyses. Download Table S4, XLSX file, 0.01 MB. Copyright © 2022 Zacharia et al. 2022 Zacharia et al. https://creativecommons.org/licenses/by/4.0/ This content is distributed under the terms of the Creative Commons Attribution 4.0 International license.

### Lipidomics analyses.

Progenesis QI (Nonlinear Dynamics, Newcastle, UK) was used for spectra deconvolution, alignment, and feature identification. Blank samples (solvents that went through the same sample preparation with no bacteria) were used to exclude artifactual mass features. Mass features which eluted at *t* of >1 min, with minimum intensity higher than 100, the lowest mean abundance in the blank, and fold change over 100 from blank, were used for analysis. Following quantile normalization, multivariate tests were carried out using Ezinfo 3.0 (Umetrics AB, Umea Sweden) and Metaboanalyst 4.0 (47). Variable importance in projection analysis was performed, and discriminant metabolic features in Δ*sepD* and Δ*csrA* strains were determined. Full-scan and MS^E^ mass spectra were acquired from all masses of 30 to 2,000 Da. Identification of mass features was carried out using 18 metabolite libraries compatible with Progenesis QI, as well as our internal library, based on mass accuracy of <5 ppm, isotope pattern, fragmentation pattern, and elution time.

### Data mining for gene expression.

Changes in transcript levels mediated by *csrA* (30) were previously reported. We mined the publicly available data sets that resulted from these studies for network-based analysis of lipid metabolism regulation by T3SS activation and *csrA* inhibition. The pathways of glycerophospholipid and terpenoid quinone pathways were adapted from the Kyoto Encyclopedia of Genes and Genomes (KEGG) database and are presented and modified to allow presentation in figures. The list of enzymes involved in E. coli lipid synthesis was extracted from the KEGG database.

### Determination of GFP fluorescence intensity.

The strains with GFP fusion were grown overnight in LB containing chloramphenicol at 37°C. The overnight cultures were subcultured (1:100) in high-glucose DMEM. IPTG was added after 3 h, and growth was continued for an additional 3 h. The cells were washed and suspended in PBS. The fluorescence of GFP was measured at 485-nm excitation and 510-nm emission wavelengths using a Spark 10M microplate reader (Tecan Trading AG, Switzerland) and normalized to their respective OD_600_s (24).

### Bacterial cell size and zeta potential measurements.

EPEC strains were subcultured in DMEM at 37°C to an OD_600_ of 0.6. Bacteria were loaded onto polylysine-coated glass slides, a coverslip was mounted on top, and the cells were visualized under a phase-contrast Axio Observer Z1 microscope (Zeiss, Germany). System control and image processing were performed with Zen pro-2012 (Zeiss). Bacterial cell size was measured using ImageJ software.

Zeta potential was measured as follows. Bacteria were centrifuged for 3 min at 16,000 × *g* and 4°C, washed, and reconstituted with 1 mL of LCMS-grade water. Zeta potential was determined using Zetasizer Nano ZS (Malvern Instruments, UK) at 25°C. Zeta potential was calculated using the Smoluchowski model. Data acquisition from 15 events was recorded for every sample, and the average zeta potential was determined from four replicates (48).

### Membrane staining.

For staining bacterial membranes, static cultures of EPEC strains were subcultured in DMEM at 37°C to an OD_600_ of 0.6. The cells were washed with PBS, treated with 1 mg/mL of FM4-64 (Molecular Probes, Invitrogen), and spotted onto poly-l-lysine-coated coverslips. Bacteria were visualized in an Eclipse Ti microscope (Nikon, Japan) equipped with a CoolSnap HQII camera (Photometrics, Roper Scientific, USA). System control, image processing, and fluorescence intensity measurement were performed with NIS Elements AR 4.3 (Nikon, Japan).

### LPS O-antigen quantification By quantitative microscopy.

For staining bacterial O antigen, static cultures of EPEC strains were subcultured in DMEM at 37°C to an OD_600_ of 0.6. Bacteria were washed with PBS and spotted onto poly-l-lysine-coated coverslips. Bacteria on the coverslips were then fixed with 2% paraformaldehyde and 0.01% glutaraldehyde in sodium cacodylate buffer (0.1 M, pH 7.2) for 15 min at 25°C. Subsequently, coverslips were washed 3 times in PBS and incubated for 30 min with 2% bovine serum albumin (BSA) and then with rabbit anti-O127 antibody (1:500, 2% BSA) for 1 h at 25°C. Coverslips were washed 3 times with PBS and incubated for 1 h at 25°C with Alexa Fluor 488-conjugated goat anti-rabbit antibody (1:1,000). Coverslips were then washed 3 times with PBS and fixed with 2.5% glutaraldehyde in sodium cacodylate buffer (0.1 M, pH 7.2) for 15 min at 25°C. Bacteria were visualized with an Eclipse Ti microscope (Nikon, Japan) equipped with a CoolSnap HQII camera (Photometrics, Roper Scientific, USA). System control, image processing, and fluorescence intensity measurement were performed with NIS Elements AR 4.3 (Nikon, Japan).

### LPS extraction and O-antigen quantification by Western blotting.

For LPS extraction, overnight-grown cultures in LB broth supplemented with antibiotics (see “Bacterial cultures” above) were subcultured in DMEM at 37°C to an OD_600_ of 0.6. The cells were centrifuged at 10,000 × *g* for 10 min, and the pellet was collected. The pellets were reconstituted in 200 μL of Laemmli buffer with bromophenol blue dye, kept at 95°C for 15 min, and allowed to cool at room temperature for 15 min. A total of 10 μL of proteinase K solution (10 mg/mL) was added to the samples and incubated at 59°C for 3 h. A total of 200 μL of ice-cold-water-saturated phenol was added to the samples. The samples were vortexed for approximately 5 to 10 s and incubated at 65°C for 15 min with occasional vortexing. After cooling to room temperature, 1 mL of diethyl ether was added to each sample, followed by vortexing (5 to 10 s). The samples were centrifuged (13,000 × *g*, 10 min). The lower blue layer was carefully removed, and samples were run on 12% SDS-polyacrylamide gels (Mini-PROTEAN TGX stain free). The bands were transferred to a 0.2 μM nitrocellulose membrane (Trans Blot Turbo; Bio-Rad Laboratories, USA). The blot was blocked with BSA and skim milk (0.6%) in Tris-buffered saline (TBS) overnight at 4°C. The membrane was then incubated with rabbit anti-O127 antibody (1:1,000) for 1 h at room temperature, washed 3 times with TBS-Tween (TBST), and incubated with anti-rabbit IgG (whole molecule)-alkaline phosphatase (AP) antibody (1:10,000) for 1 h. The membrane was then washed with TBST two times. Finally, the membrane was washed with AP buffer (100 mM Tris-HCl [pH 9.0], 150 mM NaCl, and 1 mM MgCl_2_). The membrane was visualized using Geldoc Ezimagin (Bio-Rad USA) with transwhite background.

### Vancomycin permeability assays.

Overnight-grown EPEC strains were subcultured in DMEM in a 96-well transparent flat-bottom plate. Vancomycin (Sigma-Aldrich, St. Louis, MO; 0, 3, 10, 30, and 100 μM) was incubated with EPEC strains at 37°C. The growth rate was monitored at 600 nm for 12 h using a Spark 10M microplate reader (Tecan Trading AG, Switzerland).

HeLa cells were seeded in a 24-well plate (Nunc) at a density of 7 × 10^4^ per well and grown overnight in DMEM supplemented with 10% fetal calf serum (Biological Industries) and antibiotics (penicillin-streptomycin solution; Biological Industries). They were infected with statically overnight-grown wild-type EPEC *nleA gfp* (1:100). The infection was carried out in the presence of vancomycin (30 μM). After 3 h of infection, the wells were washed with PBS twice. To terminate the infection, the cells were then fixed (3.7% formaldehyde in PBS), washed, perforated (PBS and 0.25% Triton X-100 for 10 min), washed, stained with phalloidin rhodamine (Sigma), and analyzed by fluorescence microscopy with an Axio Observer Z1 microscope (Zeiss, Germany). System control and image processing were performed with Zen pro-2012 (Zeiss).

HeLa cells were seeded in a 24-well plate (Nunc) at a density of 7 × 10^4^ per well and grown overnight in DMEM supplemented with 10% fetal calf serum (Biological Industries) and antibiotics (penicillin-streptomycin solution; Biological Industries). HeLa cells were infected with statically overnight-grown wild-type EPEC (1:100). The infection was carried out in the presence of vancomycin (30 μM). After 3 h of infection, the wells were washed with PBS twice. Triton X-100 (0.5%) was added and incubated for 10 min at room temperature. Bacteria regained from the cell surface were plated onto the LB agar and incubated at 37°C overnight. The colonies were counted and the ratio of adherent cells to planktonic cell state was determined.

### Serum bactericidal assay.

Overnight-grown EPEC strains were subcultured in DMEM in a 96-well transparent flat-bottom plate. Human serum samples were heat inactivated at 56°C. Serum samples were diluted from 1:1,250 to 1:6,250 and incubated with EPEC strains at 37°C. The growth rate was monitored at 600 nm for 12 h using a Spark 10 M microplate reader (Tecan Trading AG, Switzerland).
