# Supplementary figures and images for: Activation of the Type III Secretion System of Enteropathogenic Escherichia coli Leads to Remodeling of Its Membrane Composition and Function
Source: mSystems. 2022 Apr 28;7(3):e00202-22. doi: 10.1128/msystems.00202-22 (PMC9238428; doi:10.1128/msystems.00202-22)

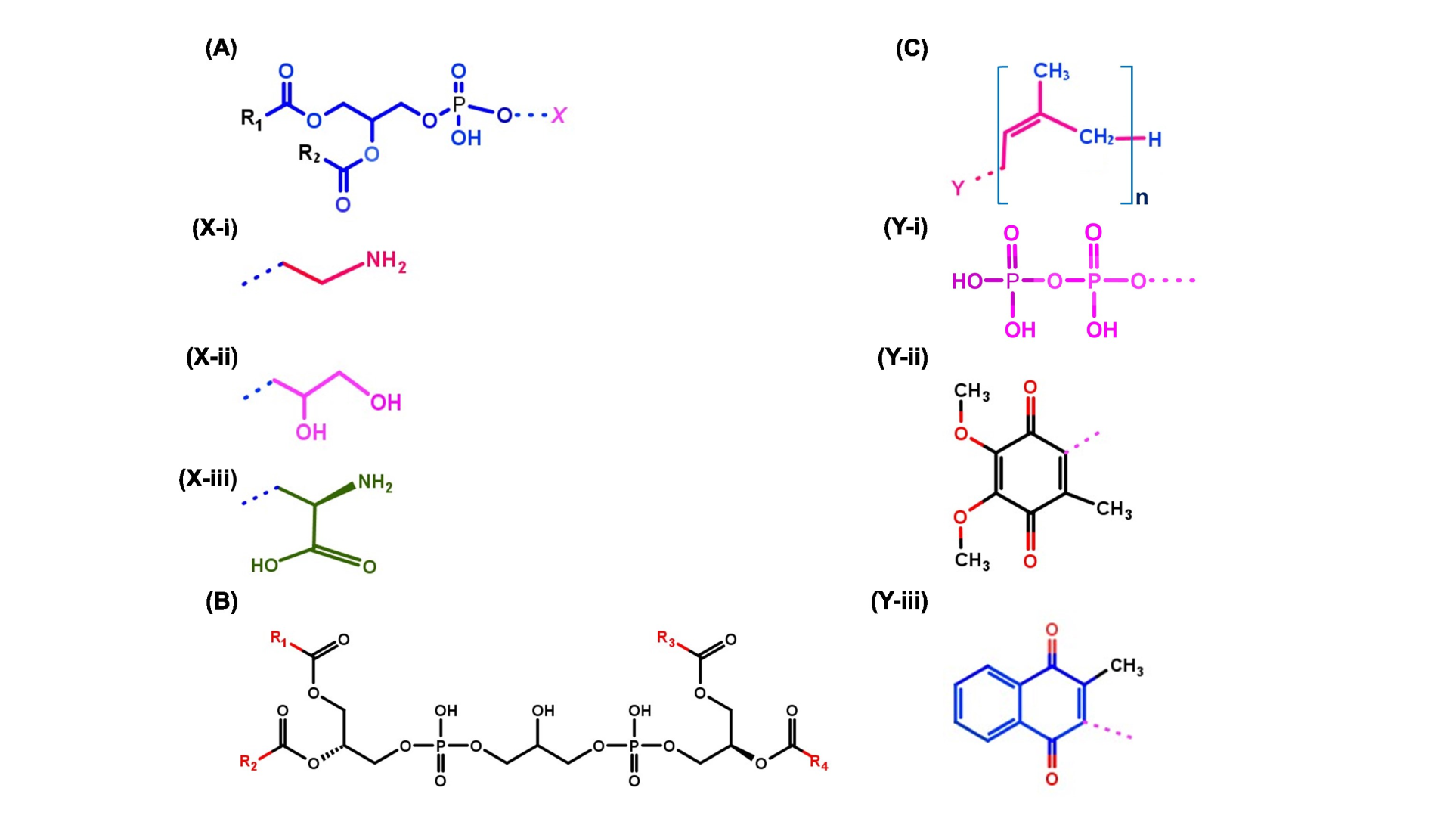

Supplement: FIG S1 [file msystems.00202-22-s0001.tif]

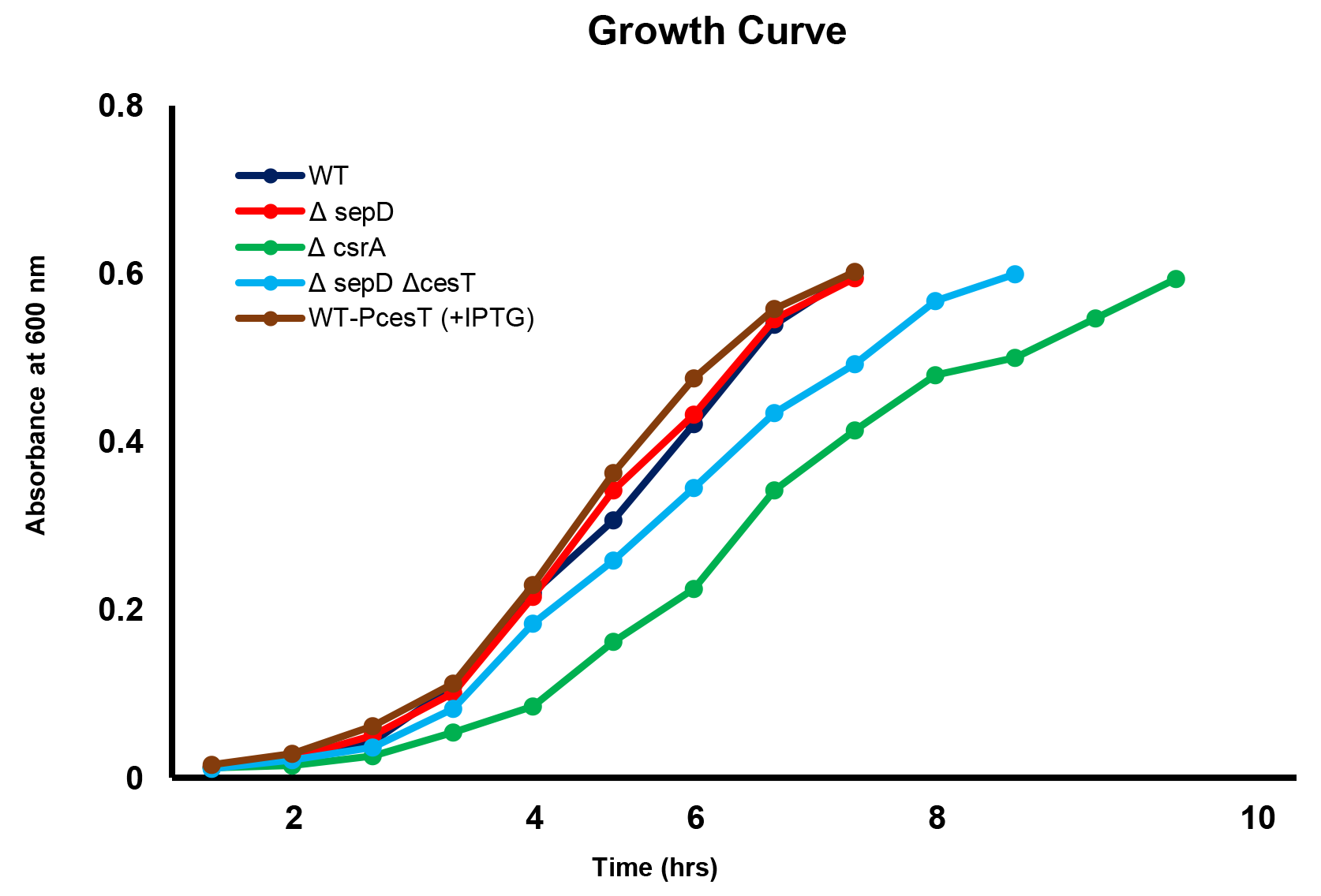

Supplement: FIG S2 [file msystems.00202-22-s0002.tif]

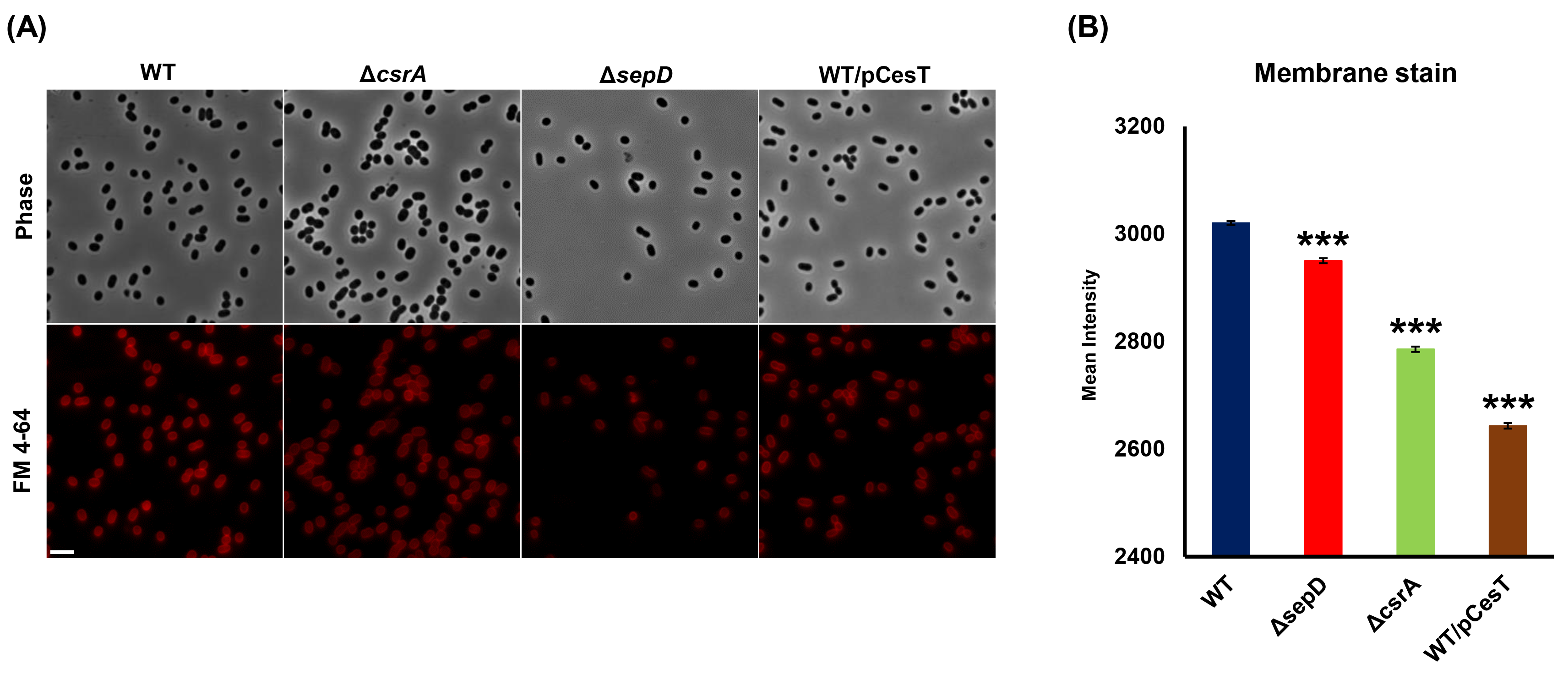

Supplement: FIG S3 [file msystems.00202-22-s0003.tif]

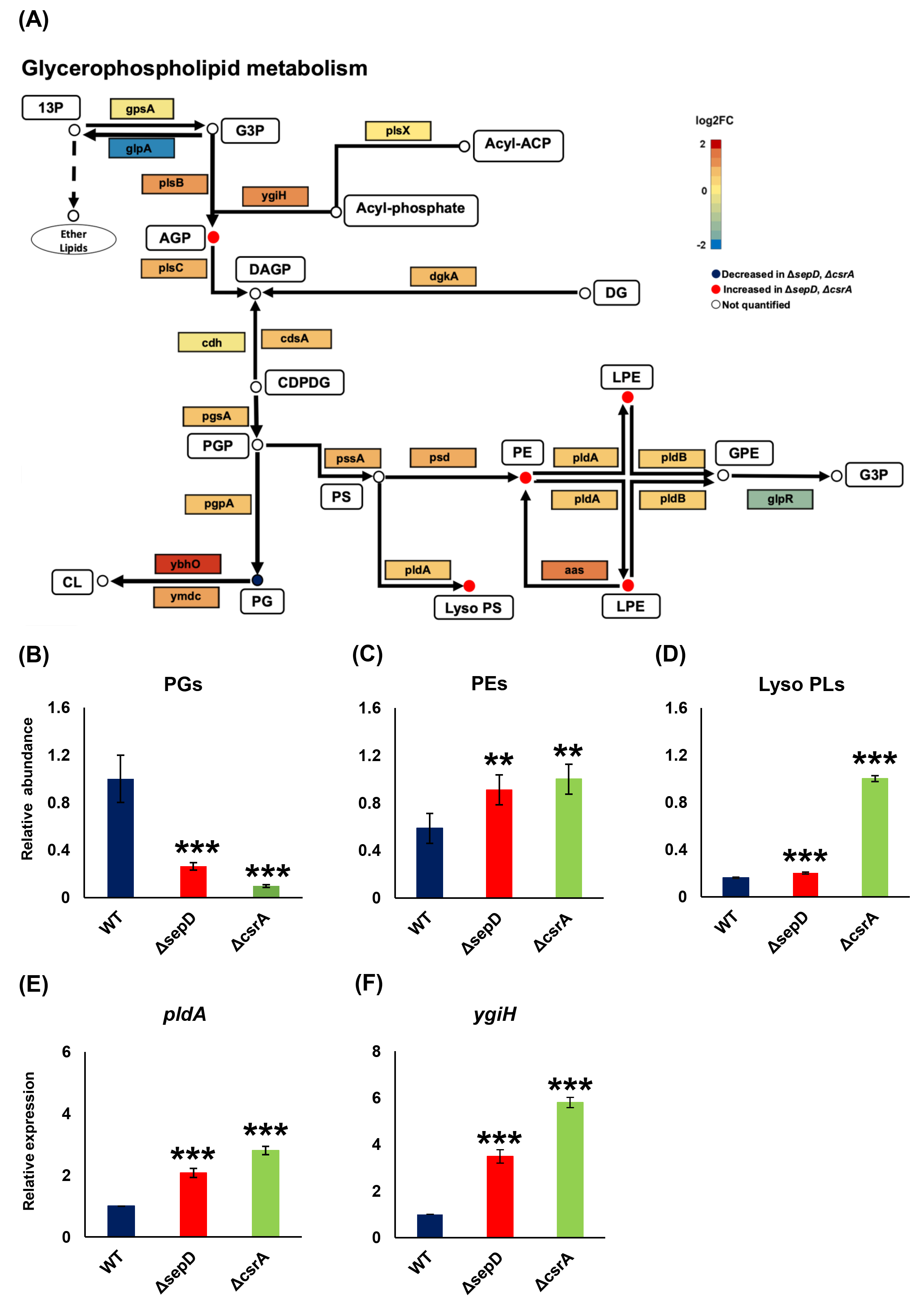

Supplement: FIG S4 [file msystems.00202-22-s0004.tif]

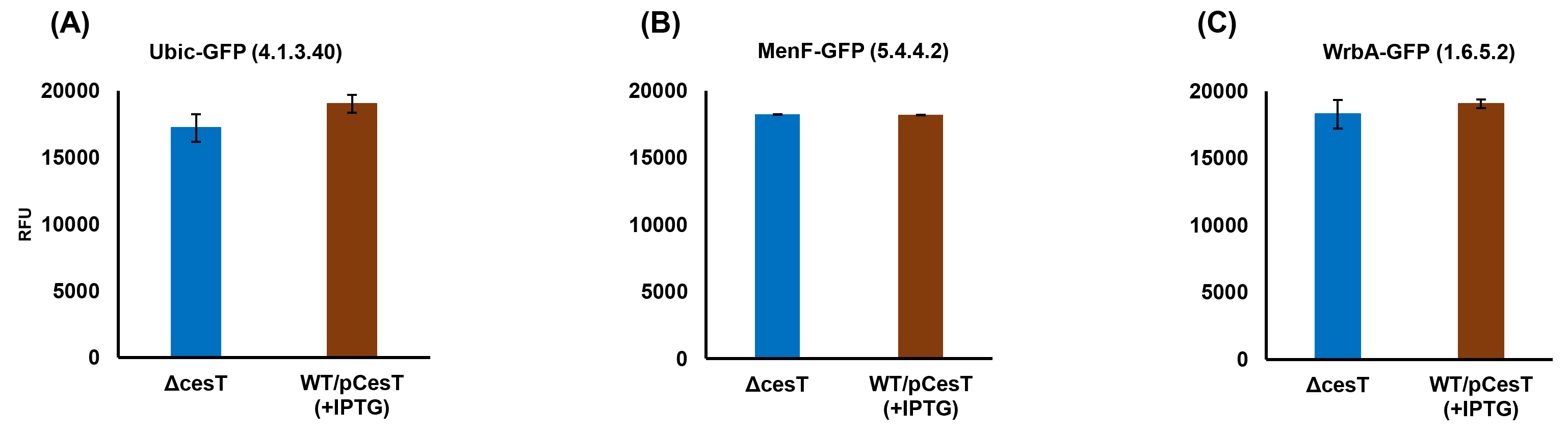

Supplement: FIG S5 [file msystems.00202-22-s0005.tif]

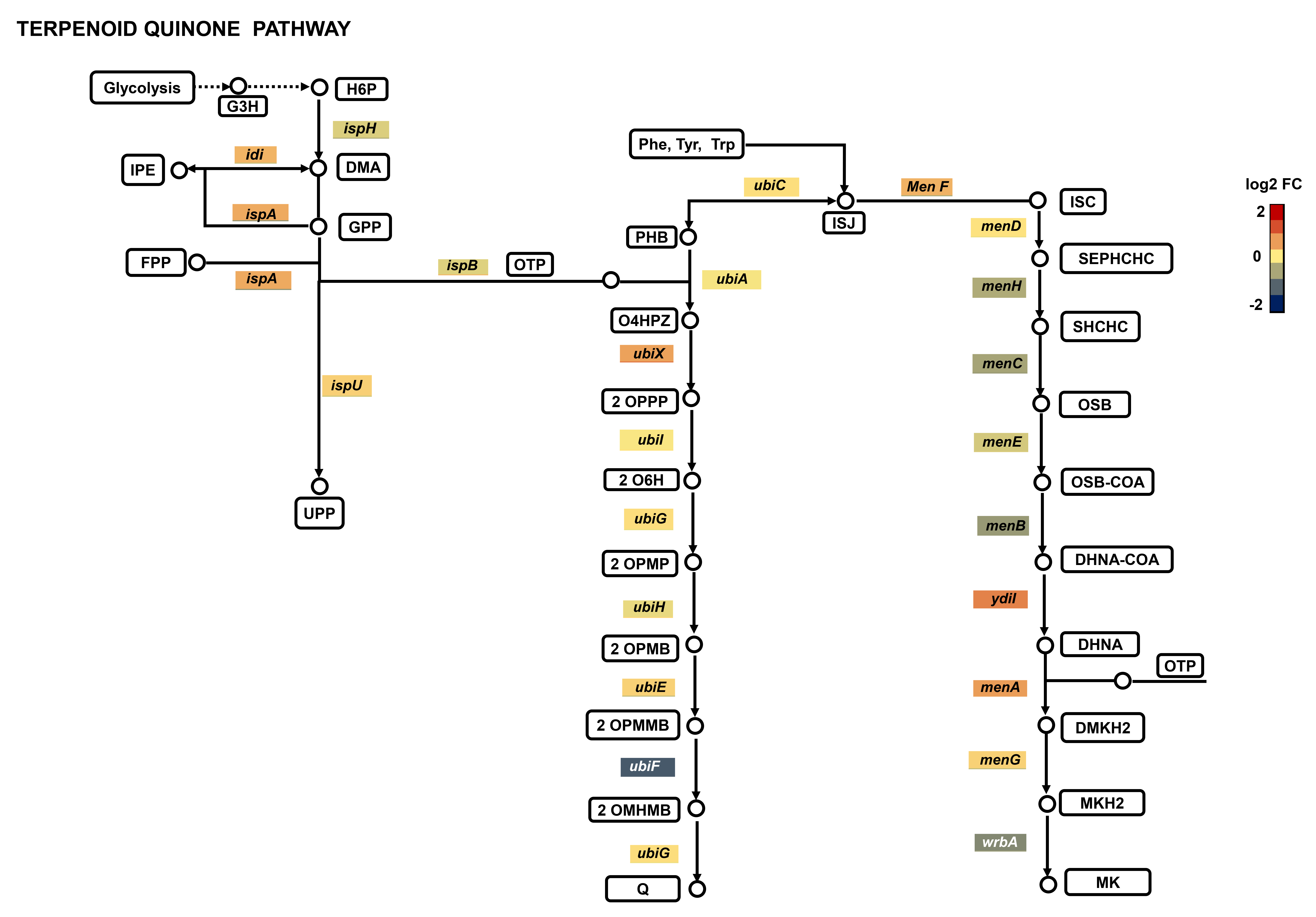

Supplement: FIG S6 [file msystems.00202-22-s0006.tif]
